# Targeted Protein Degradation: From Molecular Mechanisms to Emerging Therapeutic Strategies

**DOI:** 10.1002/mco2.71125

**Published:** 2026-11-05

**Authors:** Huaijuan Guo, Haishuang Duan, Yang Liu, Kelan Fang, Lujia Xiao, Ling Bai, Xiao Qu, Xuebing Yan, Kun Zhang

**Affiliations:** ^1^ Department of Oncology The Affiliated Hospital of Yangzhou University, Yangzhou University Yangzhou Jiangsu China; ^2^ Department of Pharmacy and Central Laboratory Sichuan Academy of Medical Sciences, Sichuan Provincial People's Hospital, School of Medicine, University of Electronic Science and Technology of China Chengdu Sichuan China; ^3^ Department of Pathology School of Medicine, Duke University Durham North Carolina USA; ^4^ Department of General Surgery The First Affiliated Hospital of USTC, Division of Life Sciences and Medicine, University of Science and Technology of China, Anhui Province Key Laboratory of Hepatopancreatobiliary Surgery Hefei Anhui Province China; ^5^ Jiangsu Provincial Innovation and Practice Base For Postdoctor Suining People's Hospital, Affiliated Hospital of Xuzhou Medical University Xuzhou Jiangsu China

**Keywords:** autophagy‐targeting chimeras, lysosome‐targeting chimeras, nanomedicine, proteolysis‐targeting chimeras, targeted protein degradation, therapeutic development

## Abstract

Targeted protein degradation (TPD) redirects endogenous protein clearance pathways to eliminate disease‐associated proteins, offering an alternative to conventional occupancy‐based inhibition. Advances in TPD have expanded the range of degradable targets from intracellular proteins to cell‐surface and extracellular proteins, although these modalities differ in their mechanisms and delivery requirements. Translating these differences into rational degrader design and effective therapeutic strategies remains challenging. This review compares proteasome‐dependent degradation, endocytosis‐mediated lysosomal degradation, macroautophagy, and chaperone‐mediated autophagy, together with representative degrader classes and target scope. We discuss molecular factors influencing degradation efficacy, including target accessibility, selection of degradation machinery, productive complex formation, degradation kinetics, and target resynthesis. We then examine strategies for improving degrader delivery and spatiotemporal control, including local administration, molecular conjugation, conditional activation, and nanotechnology. Particular attention is given to the roles of nanomaterials in improving degrader delivery, controlling drug release, and constructing functional nanoscale degradation systems. Finally, we summarize preclinical evidence and clinical advances in oncology and nononcology indications and discuss limitations and future directions for therapeutic translation. By integrating degradation mechanisms, molecular design, delivery strategies, and pharmacological considerations, this review provides a framework for selecting TPD approaches according to target biology and disease context.

## Graphical abstract

Graphical overview of targeted protein degradation: mechanisms, molecular determinants, delivery strategies, and therapeutic development. This graphical abstract summarizes the major degradation pathways involved in targeted protein degradation, including the ubiquitin–proteasome system, endocytosis–lysosome pathway, and autophagy‐dependent degradation. It further highlights key molecular determinants, nanotechnology‐based and non‐nanoparticle delivery strategies, and advances in preclinical development and clinical translation.

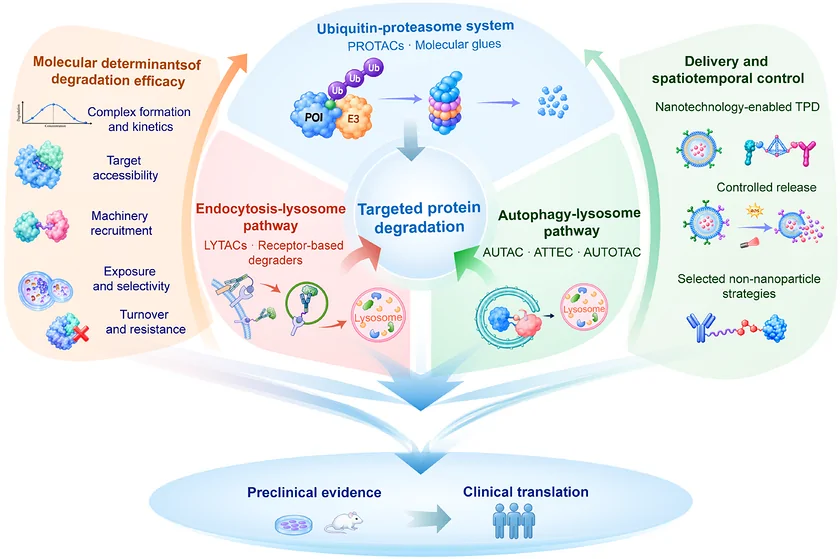

## 1 Introduction

In recent decades, substantial advancements have been made in the development of small‐molecule inhibitors targeting disease‐related proteins to exert therapeutic effects, such as the successful application of imatinib [1]. However, many disease‐relevant proteins either lack tractable small‐molecule binding pockets or exert important noncatalytic and scaffolding functions that cannot be fully suppressed by conventional occupancy‐based inhibitors [2, 3, 4]. Targeted protein degradation (TPD) provides an alternative pharmacological strategy [5]. It redirects endogenous degradation machinery to eliminate specific disease‐associated proteins rather than continuously occupying functional binding sites. The modern field of TPD can be traced to the first PROTAC proof of concept reported in 2001, in which a chimeric molecule recruited a target protein to an E3 ubiquitin ligase and induced its degradation [6]. Subsequent mechanistic studies showed that both proteolysis‐targeting chimeras (PROTACs) and molecular glue degraders exploit induced protein interactions to promote target ubiquitination and proteasomal clearance [7].

Among these degradation strategies relying on the ubiquitin–proteasome system (UPS), PROTAC has been employed to target various intracellular proteins, including BRD4, CDK4/6, BCL‐xL, and BCL‐2 [8, 9, 10]. Multiple PROTACs have entered clinical development, further supporting the therapeutic potential of TPD [10, 11, 12, 13]. However, pathogenic proteins, including certain cell surface receptors and secreted proteins, cannot be effectively degraded by traditional PROTAC technology due to their transmembrane structure or extracellular localization [14, 15]. Lysosome‐targeting chimera (LYTAC) technology addresses this limitation by utilizing the endocytosis‐lysosomal degradation pathway [16]. LYTAC molecules consist of a lysosome‐targeting ligand and a target protein‐binding component, which guides the target protein to the lysosome for degradation [17]. Notably, transmembrane proteins such as EGFR, PD‐L1, CD71, and HER2 have been successfully degraded using LYTAC technology [18, 19, 20]. Beyond proteasome‐ and receptor‐mediated lysosomal degradation, autophagy‐based strategies further extend the range of targets accessible to TPD, including selected long‐lived proteins, protein aggregates, and abnormal organelles [21, 22]. These developments have expanded TPD from intracellular proteasomal degradation toward a broader pharmacological framework capable of engaging multiple cellular protein‐clearance pathways.

Despite the expanding therapeutic potential of TPD, the physicochemical properties of many heterobifunctional degraders, including high molecular weight, polarity, and conformational complexity, can limit solubility, membrane permeability, and systemic exposure [23, 24]. These constraints have prompted strategies that regulate degrader distribution, cellular access, and activation in addition to optimizing the degrader molecule itself. Conjugation of a BRD4‐directed PROTAC to an ROR1‐targeting antibody enabled receptor‐dependent internalization and improved pharmacokinetic and antitumor performance relative to the unconjugated degrader, illustrating how molecular targeting can reshape degrader exposure [25]. Spatial control can also be introduced at the level of degrader activation. Dual‐action‐only PROTACs were designed to remain inactive unless exposed simultaneously to hypoxia and cathepsin L, thereby restricting formation of the active degrader to a defined pathological environment and reducing cytotoxicity associated with constitutive activity [26]. Nanotechnology provides an additional means of coordinating delivery with intracellular release. In a sequentially responsive CDK4/6 nano‐PROTAC, acidic conditions promoted nanostructure disassembly and tumor penetration [9]. Subsequent cathepsin B‐mediated cleavage released the active degrader intracellularly and enhanced CDK4/6 degradation compared with the free PROTAC. Together, these studies illustrate that increasing degrader exposure or accumulation alone is insufficient. Therapeutic development depends not only on achieving target degradation, but also on controlling where and when degradation occurs in vivo.

In recent years, a growing number of studies and reviews have addressed TPD, but most have focused on specific degradation modalities, particular classes of degraders, or individual delivery strategies, with limited integration of degradation mechanisms, molecular design, delivery approaches, and therapeutic translation. Accordingly, this review first summarizes the molecular mechanisms and target spectra of the major TPD modalities, and then discusses the key molecular and pharmacological factors that influence degradation efficiency. We further examine delivery and spatiotemporal control strategies, including local administration, molecular conjugation, conditional activation, and nanotechnology, with particular attention to how these approaches shape the in vivo behavior and therapeutic effects of degraders. Finally, we summarize preclinical and clinical development across TPD modalities and the major translational challenges that remain, with the aim of informing target and degradation pathway selection, optimization of degrader and delivery strategies, and the further clinical development of TPD therapeutics. A schematic overview of the scope and organization of this review is shown in Figure 1.

**FIGURE 1:**
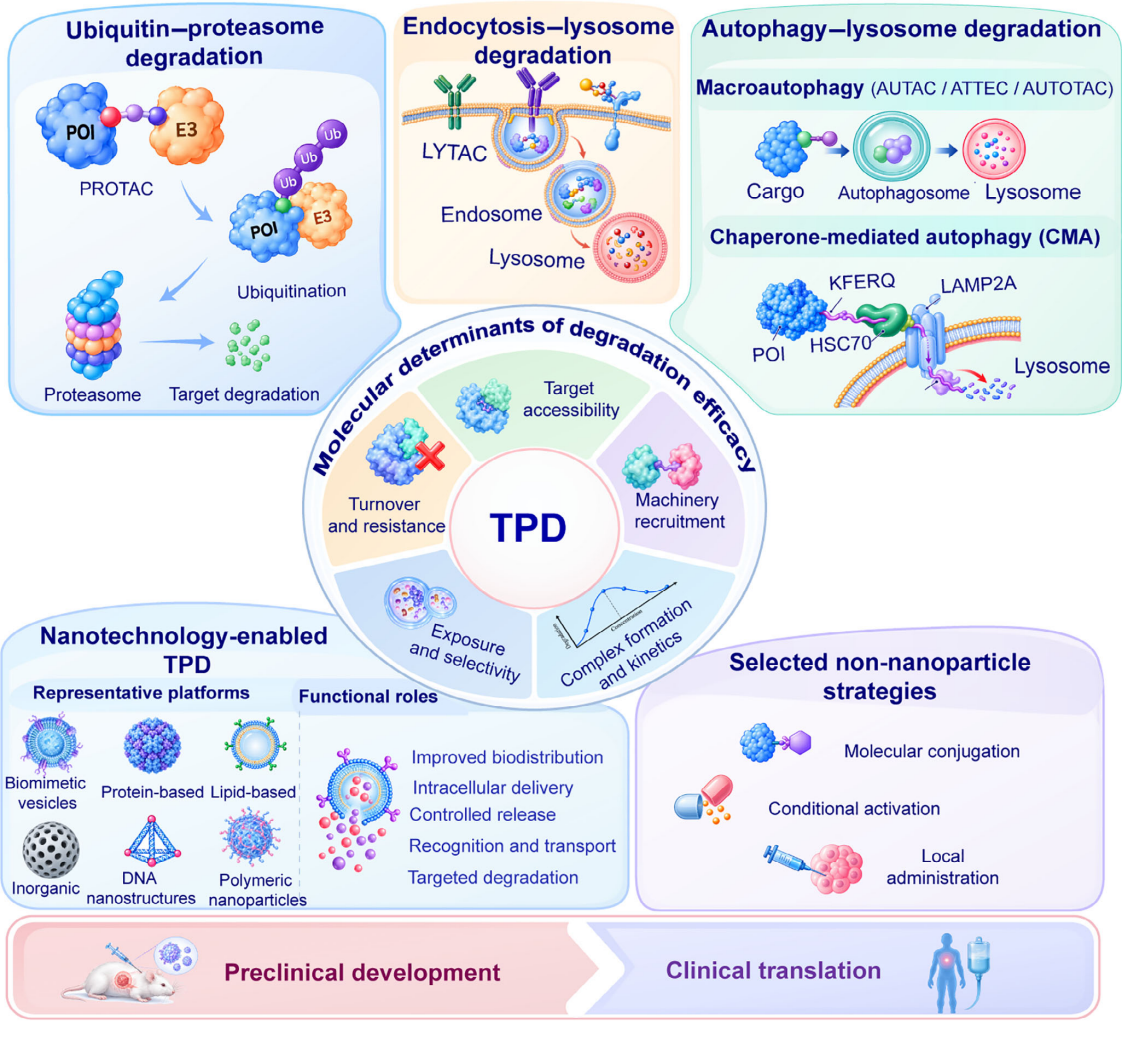
Overview of the major degradation pathways, molecular determinants, and delivery strategies involved in targeted protein degradation (TPD). The major degradation pathways include the ubiquitin–proteasome system, represented by proteolysis‐targeting chimeras (PROTACs), the endocytosis–lysosome pathway, represented by lysosome‐targeting chimeras (LYTACs), and autophagy–lysosome degradation, involving autophagy‐targeting chimeras (AUTACs) and chaperone‐mediated autophagy (CMA). Degradation efficiency is influenced by target accessibility, degradation machinery recruitment, complex formation and kinetics, effective exposure and selectivity, and target turnover and resistance. The figure also highlights representative nanomaterial platforms and their roles as delivery carriers and functional scaffolds, selected non‐nanoparticle strategies, and the progression from preclinical development to clinical translation.

## 2 Molecular Mechanisms and Target Spectrum of Protein Degradation

TPD couples selective recognition of a protein of interest (POI) to endogenous degradation pathways, resulting in removal of the bound substrate [2, 5]. Unlike enzyme inhibition, protein degradation can remove functions that depend on the presence of the entire protein, including scaffolding and signaling functions [3, 4].

However, target recognition alone does not guarantee degradation. Degradation efficiency therefore depends on productive recruitment, intracellular trafficking and the activity of the relevant degradation pathway [27, 28, 29]. In mammalian cells, the principal routes relevant to TPD are the UPS, receptor‐mediated endocytosis followed by lysosomal degradation, and lysosomal degradation through autophagy [5]. PROTACs, molecular glue degraders, LYTACs, and autophagy‐based degraders are distinct TPD modalities that use these endogenous pathways in different ways.

### 2.1 Proteasome‐Dependent TPD

Proteasome‐based TPD includes several strategies that ultimately direct intracellular proteins to proteasomal degradation [30]. Canonical approaches recruit a POI to an E3 ubiquitin ligase, leading to ubiquitination and subsequent proteasomal degradation. This principle applies to both PROTACs and molecular glue degraders, although the two classes differ in molecular architecture and in how they recruit the target to the E3 ligase [31, 32]. Alternative approaches can bypass E3 ligase recruitment by recruiting targets directly to the proteasome or to proteasome‐associated proteins [33, 34, 35, 36]. The mechanism of action of their TPD strategy is shown in Figure 2.

**FIGURE 2:**
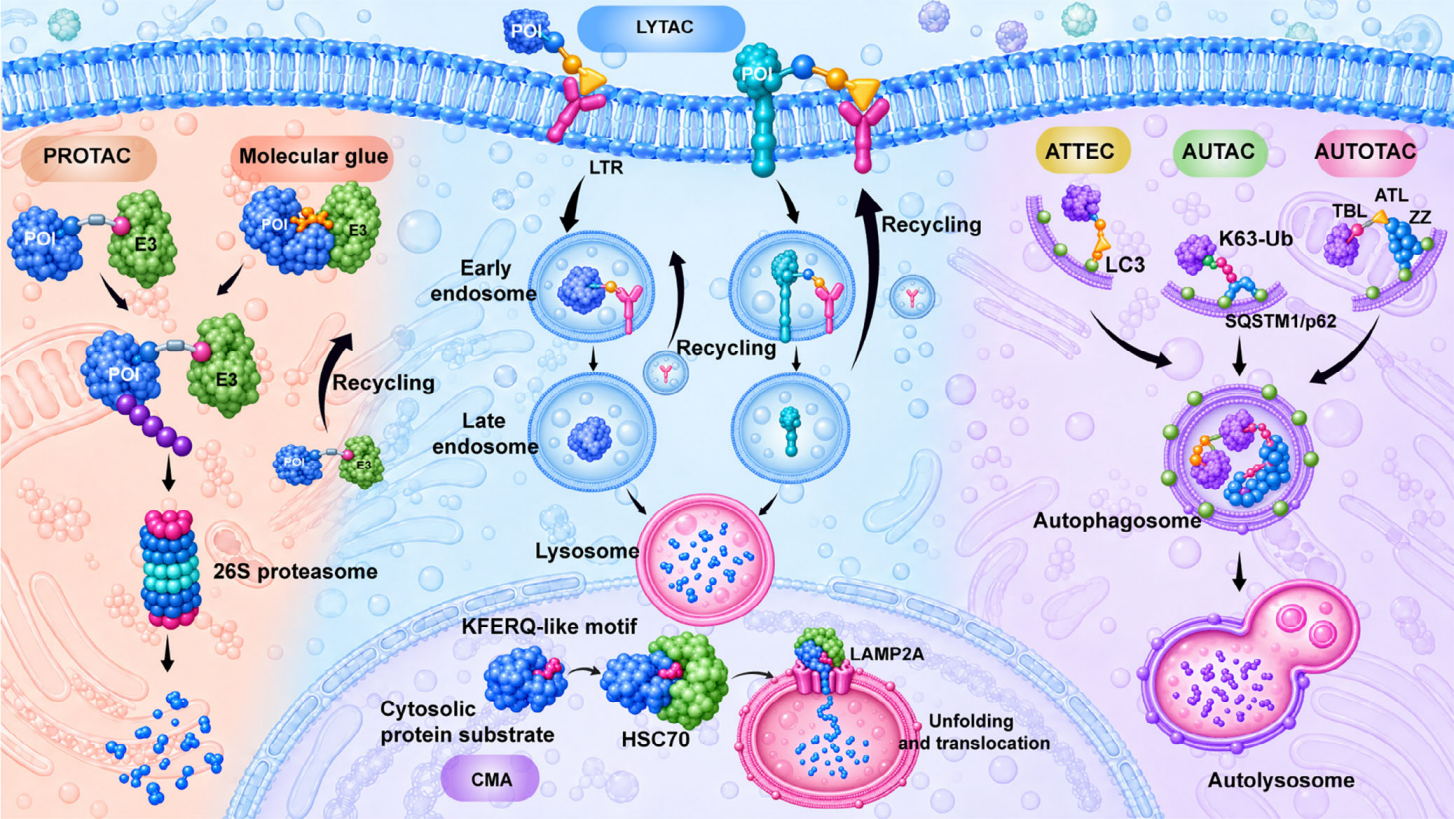
Mechanism of action of the targeted protein degradation strategy. Proteolysis‐targeting chimeras (PROTACs) and molecular glues promote E3 ligase‐mediated ubiquitination and subsequent proteasomal degradation of target proteins. Lysosome‐targeting chimeras (LYTACs) facilitate the internalization and lysosomal degradation of extracellular and membrane‐associated proteins through lysosome‐trafficking receptors. Autophagy‐based strategies, including autophagosome‐tethering compounds (ATTECs), autophagy‐targeting chimeras (AUTACs), and AUTOphagy‐TArgeting Chimeras (AUTOTACs), promote target degradation through distinct mechanisms involving LC3, ubiquitination, or p62‐mediated cargo recognition. Chaperone‐mediated autophagy (CMA) enables the selective lysosomal degradation of proteins through HSC70 recognition and LAMP2A‐mediated translocation.

#### 2.1.1 Proteolysis‐Targeting Chimeras

PROTACs are heterobifunctional molecules that recruit proteins of interest to the UPS for degradation [37, 38]. A PROTAC contains a ligand for the POI and a ligand for an E3 ubiquitin ligase connected by a linker. Simultaneous binding generates a POI‐PROTAC‐E3 ternary complex [39]. The ternary complex enables ubiquitin transfer to the target, after which the ubiquitinated protein is recognized, unfolded and degraded by the 26S proteasome [40]. The first PROTAC study demonstrated that chemically induced recruitment of a target to an E3 ligase could trigger ubiquitination and degradation [6]. Small‐molecule PROTACs subsequently achieved potent degradation in cells and target reduction in mice, moving the concept from biochemistry toward pharmacology [41]. Among the most widely used E3 recruiters in PROTAC design are cereblon (CRBN), the substrate receptor of CRL4–CRBN, and von Hippel‐Lindau protein (VHL), the substrate receptor of CRL2–VHL [2, 8, 41]. The resulting POI‐PROTAC‐E3 ternary complex can orient accessible lysine residues of the substrate toward the ubiquitination machinery, thereby facilitating ubiquitin transfer [39]. Once an appropriate ubiquitin signal has been assembled, the substrate is recognized by the 26S proteasome, unfolded and translocated into the proteolytic core for degradation. After substrate processing, a reversible PROTAC may participate in further cycles. In an early example, a RIPK2 PROTAC produced more than 95% depletion in THP‐1 cells with a DC_50_ of about 1.4 nM, showing that efficient cellular degradation is possible at low exposure [41]. Target binding alone does not establish degradability because degradation also depends on compatibility with the recruited UPS machinery [42, 43]. Cytoplasmic and nuclear proteins are prominent substrates, and membrane proteins may be tractable when they expose an intracellular domain. By contrast, soluble extracellular proteins are generally inaccessible to the cytosolic UPS and require alternative routes for degradation.

Expansion of the PROTAC target space has depended on both ligand discovery and direct measurement of degradability. Kinome‐wide chemoproteomic analyses revealed that only some proteins bound by a multi‐kinase ligand were degraded, underscoring that a binding map is not a degradability map [42, 43]. Conversely, studies of receptor tyrosine kinases found that degradation suppressed signaling for longer than matched inhibition in the systems examined [44]. Protein removal and inhibition should therefore be evaluated as distinct perturbations, with their biological consequences compared directly. Degradability can also vary among different molecular states of the same target, as illustrated by RAS‐directed degraders. Early proof‐of‐concept studies such as LC‐2 established that endogenous KRAS^G12C^ could be degraded through VHL recruitment [45]. More recent pan‐KRAS degraders have revealed a stronger dependence on target state. ACBI3 achieved broad degradation across multiple oncogenic KRAS variants but showed limited activity against KRAS^G12R^ and several Q61 mutants [46]. These resistant variants preferentially occupy the GTP loaded KRAS(On) state, which is poorly engaged by ACBI3 [47]. ACBI4 was then optimized to form highly cooperative ternary complexes with GTP‐loaded KRAS and extended degradation to these challenging KRAS(On) variants [47]. Together, these findings show that degradability is shaped not only by target identity, but also by mutation context, nucleotide state and ligand‐binding mode.

#### 2.1.2 Molecular Glue Degraders

Molecular glues represent a distinct class of targeted protein degraders that mediate protein elimination by inducing or stabilizing productive interactions between an E3 ubiquitin ligase and a POI [31]. Rather than carrying two independent binding elements as in PROTACs, these compounds promote the formation or stabilization of specific protein interfaces, enabling recruitment of a target protein to an E3 ubiquitin ligase [32]. The newly established interaction facilitates ubiquitin conjugation on the target protein, which is subsequently recognized and degraded through the proteasome pathway. Lenalidomide provides a clinically important example. By altering CRBN substrate recognition, it recruits and depletes the myeloma‐associated transcription factors IKZF1 and IKZF3 [48]. This mechanism also illustrates why the physicochemical constraints associated with large heterobifunctional PROTACs should not be directly extended to all molecular glues. Their smaller structures may favor permeability and oral exposure, although pharmacokinetic performance remains compound specific. Glue activity arises from a composite surface created by the compound and the E3 ubiquitin ligase, which enables recruitment of a complementary neosubstrate [49]. Because this induced interface rather than binary affinity alone determines productive recognition, molecular glue discovery often requires integration of cellular screening, proteomics, genetic perturbation, and structural or biophysical analysis [7]. CRBN‐recruiting ligands may also retain intrinsic molecular glue activity and induce degradation of unintended neosubstrates, which represents an additional source of off‐target degradation that should be considered during degrader development [50]. More broadly, molecular glues belong to the larger class of proximity‐inducing molecules, whereas the term molecular glue degrader specifically refers to compounds in which the induced interaction results in target degradation.

Molecular glue mechanisms extend beyond neosubstrate degradation mediated by CRBN‐binding immunomodulatory drugs [51]. Structural studies of E7820 revealed extensive DCAF15–RBM39 contacts that compensate for the low affinity of E7820 for DCAF15 [51]. By contrast, CR8 promotes association of the CDK12–cyclin K complex with the CUL4 adaptor protein DDB1, bypassing the requirement for a canonical substrate receptor [52]. Scalable chemical profiling subsequently provided a route for identifying cyclin K degraders with nonobvious recruitment mechanisms. Together, these studies emphasize that molecular glue activity depends on formation of a productive induced protein interface rather than on strong binary binding alone. Recent CRBN studies have further expanded both structural understanding and the experimentally observed neosubstrate landscape. Cryo‐electron microscopy showed that CRBN E3 ligase modulators can favor a closed CRBN conformation competent for substrate recruitment [53]. Proteomic screening identified additional CRBN neosubstrates, while structural analyses suggested that further proteins may contain compatible recognition motifs [54].

The chemistry of induced recognition is further expanded by covalent and metabolically enabled molecular glues. DCAF16‐dependent BRD9 degraders have exploited distinct cysteine‐engagement strategies, including reversible covalent chemistry, demonstrating that ligase recruitment surfaces can be chemically remodeled rather than treated as fixed attachment sites [55, 56]. A DCAF11 molecular glue targeting DDX18 is activated through glutathione conjugation, linking cellular metabolism to formation of the active recruitment interface [57]. Separately, intramolecular bivalent glues can simultaneously engage two adjacent domains of the target protein in cis, improving surface complementarity with E3 ligases and promoting productive ubiquitination and degradation [58]. These findings show that molecular glue recognition can arise through covalent engagement, metabolic activation, or target conformational reorganization, thereby extending induced‐proximity mechanisms beyond classical glue architectures.

The target spectrum of molecular glues can also be expanded by exploiting distinct protein interfaces and disease‐specific molecular dependencies. TNG961 exploited dependence on HBS1L‐mediated ribosome rescue in FOCAD‐deficient tumor models [59]. TRI‐611 recruited CRBN to ALK through a surface outside the kinase active site and showed activity in resistant and intracranial preclinical models [60]. HuR degradation altered BRAF RNA splicing in models of BRAF‐mutant colorectal cancer [49]. Together, these examples illustrate that the degradable target space of molecular glues is shaped by the compatibility among target surface features, the recruited ligase, and the biological context.

#### 2.1.3 Alternative UPS‐Dependent Strategies

Beyond conventional E3‐recruiting degraders, several UPS‐dependent strategies have been developed to expand target recognition beyond classical small‐molecule ligand‐based approaches. These approaches include hydrophobic tagging (HyT), engineered recognition‐based degraders, and antibody‐mediated degradation systems, which exploit alternative mechanisms to deliver target proteins to proteasomal degradation pathways.

HyT offers an alternative strategy for TPD that does not require the direct recruitment of a specific E3 ubiquitin ligase. HyT degraders typically consist of a target‐binding ligand (TBL) conjugated to a hydrophobic moiety through a linker [61]. Upon target engagement, the exposed hydrophobic tag can mimic structural features of misfolded proteins and activate cellular protein quality control pathways, leading to target protein elimination [62]. The initial development of HyT demonstrated that adamantane‐containing degraders could induce degradation of HaloTag fusion proteins through cellular quality‐control mechanisms [61]. Subsequent studies expanded HyT chemistry by introducing alternative hydrophobic motifs, such as norbornene, and revealed that different HyT systems may involve distinct contributions from chaperones, ubiquitination machinery, and proteasomal processing [62, 63]. Compared with conventional PROTACs, HyTs generally possess smaller molecular architectures and reduced polarity, which may provide advantages in drug‐like properties, although their degradation mechanisms remain highly dependent on the specific target and chemical design.

Engineered recognition systems provide another route to overcome the limitation that many disease‐relevant proteins lack suitable small‐molecule ligands. BioPROTACs couple engineered binding domains, such as nanobodies, with E3 ligase components to direct target proteins toward ubiquitin‐dependent degradation [64]. Trim‐Away exploits endogenous TRIM21, an antibody‐binding E3 ligase, to rapidly eliminate antibody‐bound intracellular proteins [65]. In contrast, the dTAG system utilizes a genetically introduced FKBP12^F36V^ degron, which can be recognized by heterobifunctional degraders that recruit CRBN and trigger proteasomal degradation [66]. These platforms have expanded the experimental and therapeutic accessibility of proteins that are difficult to target using conventional ligand discovery approaches by enabling degradation through engineered recognition modules.

Together, these alternative UPS‐dependent strategies broaden the degradable proteome by introducing chemical tags, engineered binders, or antibody‐based recognition systems. However, their successful translation requires consideration of endogenous target accessibility, delivery efficiency, and compatibility with cellular degradation machinery.

#### 2.1.4 Direct Proteasome Recruitment

Direct proteasome engagement expands the degradable target space to proteins that remain challenging for conventional E3 ligase recruitment strategies. This approach provides an alternative TPD pathway that does not rely on conventional E3 ligase recruitment [30]. In the canonical UPS, ubiquitin chains facilitate recognition of substrates by the 26S proteasome, followed by substrate unfolding and translocation into the 20S catalytic core for degradation. By physically linking POIs to proteasome‐associated components, direct recruitment strategies provide an alternative route for substrate engagement and processing.

One of the first demonstrations of this theory used a macrocyclic ligand for PSMD2/RPN1, a component of the 19S regulatory particle [30]. Linking this ligand to a BET‐binding moiety boosted recruitment and degradation of BRD4, establishing proteasomal subunits themselves as chemically accessible anchors for TPD. This concept was subsequently expanded through Proteasome Cap Targeting Chimeras (CAP‐TACs), which recruit POIs directly to the 19S cap proteins RPN13 or RPN1 [33]. These molecules promote ubiquitination‐independent, proteasome‐dependent degradation of targets including BRD4, PRMT5, and FKBP12. The proteasome‐associated deubiquitinase USP14 has also been exploited as a recruitment handle. USP14‐directed degraders have been reported to reduce HDAC1, IMPDH2, and CERT1, further confirming the practicality of directing structurally distinct proteins directly to the 26S proteasome [34, 35]. A related approach is CANDDY (chemical knockdown with affinity and degradation dynamics), which uses degradation tags derived from modified proteasome‐binding scaffolds [36]. The KRAS‐directed molecule TUS‐007 was reported to degrade KRAS mutants (G12D and G12V) and wild‐type KRAS, illustrating the potential of proteasome‐directed degradation for oncogenic proteins that are difficult to eliminate through conventional inhibition. Together, these approaches establish direct proteasome recruitment as an alternative strategy for target degradation that can bypass conventional E3 ligase recruitment.

### 2.2 Endocytosis‐Dependent Lysosomal Degradation

Endocytosis‐dependent lysosomal degradation extends TPD beyond intracellular substrates by enabling the elimination of extracellular and cell‐surface proteins [14]. These methods typically utilize cell surface receptors or membrane‐bound E3 ubiquitin ligases to facilitate target internalization and subsequent delivery to lysosomes [67, 68, 69]. These approaches can be grouped into conventional LYTACs, variants that exploit alternative internalizing receptors, and platforms that recruit membrane E3 ubiquitin ligases.

#### 2.2.1 Lysosome‐Targeting Chimeras

LYTACs represent a lysosome‐dependent TPD strategy that expands protein degradation beyond intracellular substrates accessible to the UPS by redirecting extracellular and membrane‐associated proteins to the endolysosomal pathway [16]. Unlike PROTACs and molecular glues, LYTACs exploit lysosome‐targeting receptors (LTRs) on the cell surface to mediate receptor‐dependent internalization and lysosomal delivery. A typical LYTAC consists of a target‐binding module linked to a lysosome‐shuttling moiety, enabling formation of a target protein–LYTAC–LTR complex that undergoes endocytosis and subsequent degradation.

The first‐generation LYTAC platform utilized the cation‐independent mannose‐6‐phosphate receptor (CI‐M6PR), which naturally mediates transport of mannose‐6‐phosphate (M6P)‐modified proteins to lysosomes [14, 28]. By conjugating M6P motifs to TBLs, extracellular proteins were redirected to CI‐M6PR‐mediated uptake and degradation. To improve tissue specificity, second‐generation LYTACs employed the hepatocyte‐specific asialoglycoprotein receptor (ASGPR) through high‐affinity binding of tri‐valent N‐acetylgalactosamine (tri‐GalNAc) [17]. In the original study, a cetuximab‐based GalNAc–LYTAC (Ctx–GalNAc) promoted ASGPR‐dependent internalization and lysosomal degradation of EGFR in hepatocellular carcinoma cell lines, including HEP3B, HEPG2, and HUH7 [17]. Moreover, Ctx–GalNAc selectively depleted cell‐surface EGFR in ASGPR‐positive HEP3B cells but not in ASGPR‐negative HeLa cells, demonstrating receptor‐dependent cell selectivity.

Beyond CI‐M6PR and ASGPR, additional endocytic receptors have expanded the target and tissue scope of LYTACs. Integrin α3β1‐mediated LYTACs using RGD ligands enabled degradation of membrane proteins such as CD71 and PTK7 and suppressed tumor growth [70, 71]. Folate receptor‐, transferrin receptor‐, and scavenger receptor A1‐based systems further broadened LYTAC applications by targeting proteins including EGFR, PD‐L1, CD20, CD47, and c‐Met [72, 73, 74]. GPC3‐mediated LYTACs have also been developed for selective degradation of tumor‐associated membrane proteins in hepatocellular carcinoma [75].

LYTACs expand the degradable proteome from intracellular proteins to extracellular and membrane‐associated targets through receptor‐mediated endocytosis and lysosomal trafficking. However, effective degradation depends not only on receptor expression but also on productive internalization, endosomal sorting, and lysosomal delivery, which remain key determinants for further development of lysosome‐dependent TPD strategies.

#### 2.2.2 Small‐Molecule and Cytokine Receptor‐Directed Variants

Subsequent extracellular TPD strategies expanded both the receptor repertoire and the molecular architectures used to access the endolysosomal pathway. These variants preserve the fundamental principle of receptor‐mediated internalization followed by lysosomal proteolysis, while introducing alternative targeting modules and endogenous trafficking receptors to broaden cellular accessibility and therapeutic applications.

Molecular degraders of extracellular proteins through the ASGPR (MoDE‐As) represent a bifunctional small molecule extension of LYTAC, demonstrating that extracellular proteins can be eliminated without antibody‐based recognition modules [76]. MoDE‐As are bifunctional molecules consisting of a TBL and an ASGPR‐engaging moiety, enabling ternary complex formation and hepatocyte‐specific internalization. Following ASGPR‐mediated internalization, the ternary complex is trafficked to lysosomes for target degradation. Cytokine receptor‐targeting chimeras (KineTACs) combine a target‐binding arm with a cytokine arm that engages its cognate cytokine receptor to redirect extracellular and cell surface proteins toward lysosomal degradation [77]. The platform introduced by Pance and colleagues used CXCL12 to engage CXCR7 and direct cell surface and extracellular proteins to the lysosome for degradation. Additional KineTACs incorporating CXCL11, vMIP‐II, and IL‐2 further demonstrated the modularity of cytokine receptor‐mediated lysosomal degradation strategies. Beyond cytokine receptor‐based systems, additional strategies have explored alternative cell‐surface receptors and transport proteins to expand the routes available for lysosomal degradation. Glucagon‐like peptide‐1 receptor (GLP‐1R) and glucose transporter 1 have recently been identified as alternative lysosomal transport receptors for selective protein degradation [78, 79]. GLP‐1R‐mediated LYTACs (g‐LYTACs) enabled degradation of extracellular and membrane proteins, including EGFR, PD‐L1, and integrins, and improved antitumor immune responses in mouse models by promoting PD‐L1 depletion [79]. Similarly, GLUT targeting chimeras (GTACs) promoted lysosomal degradation of extracellular and membrane proteins, including TNF‐α and HER2. Compared with the parent antibody, GTACs showed stronger inhibition of tumor cell growth in vitro and enhanced tumor targeting in a mouse tumor model [80]. Together, these platforms broaden the receptor repertoire available for extracellular and membrane protein degradation [81].

#### 2.2.3 Membrane E3 Ligase‐Recruiting Degradation Platforms

Whereas LYTACs and related approaches exploit internalizing receptors to transport bound cargo toward lysosomes, an alternative strategy repurposes transmembrane E3 ubiquitin ligases to remove cell‐surface proteins. Membrane E3 ligase recruiting platforms exploit ubiquitination‐dependent receptor trafficking rather than classical cytosolic proteasomal degradation. Representative platforms include antibody‐based PROTACs (AbTACs), proteolysis‐targeting antibodies (PROTABs), and receptor elimination by E3 ubiquitin ligase recruitment (REULR), with the latter using modular nanobody fusions rather than full‐length bispecific IgGs [67, 82, 83].

The AbTAC concept was initially demonstrated by Cotton et al. using AC‐1, a bispecific IgG that combines the PD‐L1‐binding arm of atezolizumab with a ring finger protein 43 (RNF43)‐binding antibody selected by phage display [68]. The two half‐antibodies were assembled through knobs‐into‐holes engineering, enabling simultaneous recognition of PD‐L1 and RNF43. In MDA‐MB‐231 cells, AC‐1 reduced PD‐L1 with a DC_50_ of 3.4 nM and a maximum degradation of 63% after 24 h. Neither individual antibody arm nor the two unconjugated antibodies reproduced this activity. RNF43 knockdown abolished PD‐L1 loss, whereas bafilomycin A1, but not MG132, suppressed degradation, establishing that the initial AbTAC acts through an RNF43‐dependent lysosomal pathway. Whole‐cell proteomic analysis detected no broad proteomic disruption, and PD‐L1 degradation was also observed in HCC827 lung cancer and T24 bladder cancer cells, supporting the portability of the approach across different cellular backgrounds.

PROTABs subsequently extended this principle through systematic evaluation of membrane E3 ligases, target receptors, and antibody formats. Marei et al. first paired antibodies against RNF43 or zinc‐ and ring finger 3 (ZNRF3) with several insulin‐like growth factor (IGF)1R‐binding antibodies and showed that the resulting bispecific molecules induced receptor ubiquitination and clearance across colorectal cancer cell lines [69]. Disruption of the ZNRF3 RING domain or inhibition of ubiquitin activation with MLN7243 impaired IGF1R degradation, directly linking PROTAB activity to E3 catalytic function. Both bafilomycin A1 and MG132 reduced degradation, indicating that ubiquitinated receptors can engage lysosomal and proteasomal machinery in a target‐ and cellular‐context‐dependent manner. Notably, efficient IGF1R clearance was retained in cells with an IGF1R:ZNRF3 surface ratio exceeding 400:1, supporting an event‐driven process in which one ligase molecule may act on multiple target receptors rather than a strictly stoichiometric mechanism. Subsequent work broadened the target scope and clarified PROTAB trafficking. Monroy et al. engineered a bispecific antibody degrader comprising a pan‐selective human αv antibody arm and an anti‐ZNRF3 arm [84]. In HepG2 cells, 10 nM of this degrader removed 69.3 ± 1.2% of cell‐surface αvβ5 and reduced total αv protein by 53% after 48 h, indicating that this strategy can be applied to large, multisubunit integrin receptors. He et al. subsequently reported approximately 85% HER2 depletion within 24 h using a HER2–ZNRF3 PROTAB [83]. Degradation was predominantly lysosomal, whereas ZNRF3 was not codegraded and the antibody was largely recycled, indicating distinct intracellular fates for the target, recruited E3 ligase, and degrader.

Building on the same mechanistic principle, the REULR platform replaced full‐length bispecific IgGs with modular nanobody fusions. REULRs were generated against five plasma‐membrane‐associated RING E3 ligases—RNF128, RNF130, RNF167, RNF43, and ZNRF3—and redirected these ligases toward EGFR, erythropoietin receptor, and PD‐1 [67]. Treatment with selected PD‐1‐directed REULRs at 50 nM produced robust, near‐complete loss of cell‐surface PD‐1 in engineered HEK293T cells, whereas the individual nanobody components were inactive. The smaller and readily reconfigurable scaffold broadens the range of usable E3–target combinations, although rapid renal clearance may limit the systemic exposure of unmodified nanobodies.

This mechanism distinguishes membrane E3‐recruiting degraders from LYTACs. LYTACs can remove both soluble extracellular proteins and cell‐surface proteins, whereas the experimentally established target spectrum of AbTACs and PROTABs is currently confined mainly to transmembrane proteins whose intracellular regions can be ubiquitinated. REULR has a similarly restricted target scope. It has been validated against the cell‐surface receptors EGFR, EpoR, and PD‐1, as well as membrane E3 ligases targeted through “fratricide” REULR constructs. Conversely, membrane E3‐recruiting platforms can be produced as fully recombinant antibody‐based molecules and may exploit the disease‐ or tissue‐restricted expression of individual E3 ligases. This creates an opportunity to combine antibody selectivity and favorable systemic exposure with context‐dependent degradation, while removing the entire receptor rather than merely blocking one functional domain

### 2.3 Autophagy‐Dependent Lysosomal Degradation

In contrast, autophagy—an essential intracellular degradation pathway—plays a pivotal role in regulating protein turnover and maintaining cellular homeostasis. Unlike LYTAC, which primarily targets extracellular or membrane‐associated proteins for lysosome‐based degradation, autophagy involves the sequestration of substrates into autophagosomes, which then fuse with lysosomes for degradation [85]. Macroautophagy can process soluble proteins, protein aggregates, and larger cellular structures, including damaged mitochondria [85]. Autophagy can also contribute to the clearance of misfolded and aggregation prone proteins, a feature relevant to several neurodegenerative diseases [86].

#### 2.3.1 Autophagy‐Targeting Chimeras

Autophagy‐targeting chimeras (AUTACs) are heterobifunctional small molecules that chemically label intracellular cargo for selective autophagy, thereby enabling the lysosomal clearance of both proteins and organelles [87]. AUTACs comprise three key components: a guanine derivative‐based degradation tag, a chemical linker, and a ligand that selectively binds the target protein or organelle. The TBL recognizes the cargo, while the guanine‐derived degradation tag mimics S‐guanylation and promotes K63‐linked polyubiquitination of the substrate [87]. This ubiquitin chain is recognized by the autophagy receptor SQSTM1/p62, which facilitates the recruitment of the ubiquitinated substrate to the autophagosomal membrane via interaction with LC3 [88].

This modular design was initially validated using first‐generation AUTACs targeting methionine aminopeptidase 2 (MetAP2), FKBP12, BET proteins, and mitochondria. Protein‐directed AUTACs included fumagillol‐based AUTAC1, synthetic FKBP ligand‐based AUTAC2, and JQ1‐derived AUTAC3, which target MetAP2, FKBP12, and BET proteins including BRD4, respectively [87]. In HeLa cells, 1–100 µM AUTAC1 reduced endogenous MetAP2 after 24 h, whereas 10 µM AUTAC2 significantly reduced FKBP12 and increased its K63‐linked polyubiquitination. By contrast, 10 µM AUTAC3 only modestly reduced BRD4 after 24 h in A549 cells, demonstrating feasibility but limited degradation potency. AUTAC4 extended the approach to organelles by linking a translocator protein ligand to the guanine‐derived tag, thereby targeting the outer mitochondrial membrane. In Down syndrome‐derived fibroblasts, 3 days of AUTAC4 treatment cleared fragmented mitochondria and restored mitochondrial membrane potential and ATP production. This mitophagy required ubiquitination but was independent of PINK1 and Parkin, supporting engagement of a noncanonical pathway.

Unlike first‐generation AUTACs, whose guanine‐derived tags contained an l‐cysteine substructure, second‐generation AUTACs (2G‐AUTACs) optimized tag chemistry without changing ligand‐directed cargo recognition or the selective autophagy mechanism [22]. Structure–activity relationship studies showed that the guanine core remained essential, whereas l‐cysteine could be replaced with aromatic heterocycles. Among these, the pyrazole‐containing derivative tt44 showed the strongest activity. Transferring the optimized tag to an FKBP12 ligand through a PEG5 linker generated a degrader active at 1 µM and approximately 100‐fold more active than AUTAC2. Several related derivatives achieved submicromolar degradation activity, and FKBP12 degradation remained p62‐dependent.

The AUTAC strategy has also been extended to SHP2. SA‐8 combines the SHP2 inhibitor SHP099 with an LC3 recruiting ligand and induces dose‐ and time‐dependent SHP2 degradation in HeLa cells (DC_50_ = 3.22 ± 0.18 µM; *D*_max_ = 80.47 ± 1.14%). Its activity was reduced by inhibitors of autophagy and lysosomal function, supporting degradation through the autophagy–lysosome pathway [89].

#### 2.3.2 Autophagosome‐Tethering Compounds

Autophagosome‐tethering compounds (ATTECs) are small molecules that simultaneously engage a target and the autophagosome‐associated protein LC3, thereby tethering the target to autophagosomal membranes for sequestration and subsequent lysosomal degradation through macroautophagy [90, 91]. ATTECs are defined by this tethering mechanism and do not necessarily adopt the two‐warhead‐and‐linker architecture typical of PROTACs. Direct recruitment of LC3 also separates them mechanistically from guanine‐tagged AUTACs and from AUTOphagy‐TArgeting Chimeras (AUTOTACs), which recruit and activate an autophagy receptor. The founding compounds bound mutant huntingtin and LC3 and preferentially lowered the mutant protein, suggesting that a disease‐associated structural state can be used to bias clearance [90].

Subsequent bifunctional ATTECs extended LC3‐mediated tethering beyond individual proteins to nonprotein cellular structures. Lipid‐droplet‐targeting ATTECs promoted selective lipophagy, whereas mitochondria‐targeting variants subsequently induced mitophagy and depletion of whole mitochondria [91, 92, 93]. This expansion requires a clear distinction between loss of an individual molecular component and clearance of the targeted cellular structure. LC3‐directed tethering has expanded from aggregation‐prone substrates to signaling proteins and adaptable recognition scaffolds [94, 95]. BCR–ABL‐directed ATTECs linked the BCR–ABL inhibitor dasatinib to the LC3B‐binding ligand GW5074, whereas 2,4‐quinazolinedione‐derived LC3B recruiters conjugated to the CDK9 inhibitor SNS‐032 enabled simultaneous autophagic degradation of CDK9 and its associated cyclin T1 [94, 95]. Covalent aptamer‐based autophagosome‐tethering chimeras use target‐binding DNA aptamers conjugated to an LC3 ligand to promote the autophagic degradation of cell‐surface proteins, including transferrin receptor 1 (TfR1) and nucleolin [96]. RNA aptamer scaffolds have also been engineered as target‐recognition and autophagy‐recruitment modules, thereby directing protein cargos to the autophagy–lysosome pathway [97]. PDEδ‐directed ATTECs further extended this strategy to a prenyl‐protein chaperone implicated in KRAS trafficking. Compound 12c induced lysosome‐dependent autophagic degradation of PDEδ, with a DC_50_ of 1.7 µM and a *D*_max_ of 85%, and inhibited the proliferation of KRAS‐mutant Capan‐1 and MiaPaCa‐2 pancreatic cancer cells more effectively than the corresponding PDEδ inhibitor [98]. It is essential to differentiate the coclearance of complex‐related components from the direct ligand recognition of each impacted protein.

Efficient ATTEC degradation requires simultaneous engagement of the target and LC3 together with a functional autophagy–lysosome pathway [99, 100]. Increased LC3‐II or colocalization with LC3 puncta alone does not establish increased autophagic flux; lysosomal turnover should also be demonstrated [92]. For mitochondria, measurements of mitochondrial mass, multiple mitochondrial proteins, and mtDNA, together with pH‐sensitive mitophagy reporters or electron microscopy, can distinguish whole‐organelle clearance from selective loss of individual mitochondrial components [92]. Organelle‐directed tethering exposes additional distinctions within lysosomal routing. TSPO–LC3 chimeras induced mitochondrial clearance in disease‐relevant cellular and organoid models, and engineered recruitment of LC3 or ATG16L1 was explored for aggregate and mitochondrial elimination [101]. An artificial mitophagy system recruiting ATG16L1 could also operate through an ATG5‐independent endosomal route [101]. Hence, recruitment of an autophagy‐associated protein is insufficient to establish canonical macroautophagy. Pathway assignment requires testing cargo enclosure, essential factors, and terminal lysosomal degradation in the particular construct.

#### 2.3.3 AUTOTACs

Fare bifunctional small molecules that promote TPD through p62/SQSTM1‐mediated selective autophagy [102]. Classical AUTOTACs consist of a TBL, a linker, and an autophagy‐targeting ligand (ATL) [103]. The TBL recognizes the POI or a pathological protein aggregate, whereas the ATL engages the ZZ domain of p62 and induces its conformational activation. Importantly, AUTOTACs do not merely tether a cargo to p62. They also convert p62 into an active, cargo‐clustering state that is competent for autophagic delivery. The recruited cargo is subsequently routed to the p62–LC3‐dependent autophagy–lysosomal pathway [104]. AUTOTACs differ mechanistically from ATTECs and AUTACs. ATTECs directly stabilize interactions between a target and LC3 and do not require p62 or a classical two‐ligand‐linker architecture. AUTACs instead contain a guanine‐derived degradation tag that mimics S‐guanylation, promotes K63‐linked polyubiquitination, and facilitates recognition by autophagy receptors such as p62. In contrast, classical AUTOTACs directly recruit and activate p62, and target degradation can proceed without target ubiquitination.

The TBL determines substrate selectivity and may be derived from inhibitors, receptor ligands, covalent ligands, chemical chaperones, or aggregate‐selective binders. AUTOTACs have been developed against estrogen receptor beta, androgen receptor (AR), and MetAP2, as well as mutant desmin, aggregation‐prone proteins, mutant huntingtin, and α‐synuclein aggregates [103, 105, 106]. The ATL is typically designed to engage the p62 ZZ domain. Ligand binding promotes p62 oligomerization through its PB1 domain and enhances LC3 engagement through the LC3‐interacting region [107]. The resulting target–AUTOTAC–p62 complex is recruited to the phagophore, enclosed within autophagosomes, and ultimately degraded after lysosomal fusion [108]. PEG‐based linkers are commonly incorporated into AUTOTACs to provide flexible spacing between the two ligands and can improve water solubility and cellular permeability. Although AUTOTAC activity can be retained across a range of linker lengths, linker architecture remains an important determinant of degradation efficiency. In the AR‐targeting series, ATC‐323, ATC‐324, and ATC‐325 contained progressively longer PEG linkers spanning three to five units. At 5 µM, ATC‐324 and ATC‐325 efficiently activated p62‐dependent autophagy and promoted AR degradation, whereas ATC‐323 showed little activity. Notably, ATC‐324 achieved an AR degradation DC_50_ of 2.05 µM and reduced AR levels by more than 50% within 6–12 h, highlighting the need for target‐specific optimization of linker length and composition [105].

AUTOTACs are particularly suited to aggregated or otherwise difficult‐to‐clear proteins. ATC161 selectively depleted aggregated α‐synuclein without substantially reducing the monomeric protein and decreased aggregate burden in an α‐synuclein preformed‐fibril mouse model [108]. AUTOTACs may also extend ligand binding into target elimination. For example, ATC‐324 degraded multiple AR ligand‐binding‐domain mutants and indirectly reduced AR‐v7 through AR‐FL/AR‐v7 heterodimerization.

#### 2.3.4 Chaperone‐Mediated Autophagy

Chaperone‐mediated autophagy (CMA) is a selective lysosomal degradation pathway that directly translocates soluble cytosolic proteins across the lysosomal membrane [109]. Canonical CMA substrates contain a KFERQ‐like motif that is recognized by cytosolic HSC70 [110]. The substrate HSC70 complex is then delivered to lysosome‐associated membrane protein 2A (LAMP2A), which functions as the lysosomal receptor for CMA substrates [111]. Substrate binding promotes the dynamic assembly of LAMP2A into a high‐molecular‐weight translocation complex that mediates passage across the lysosomal membrane [112]. Unlike macroautophagy, CMA does not require autophagosome formation. Canonical CMA substrates are soluble cytosolic proteins that contain or expose a KFERQ‐like motif recognized by HSC70 and are delivered to LAMP2A at the lysosomal membrane [113, 114]. Substrates must unfold before translocation, which limits canonical CMA to proteins compatible with direct passage across the lysosomal membrane and excludes intact organelles or large protein aggregates [86].

CMA‐based degraders exploit the substrate‐recognition machinery of CMA to redirect noncanonical proteins to lysosomes. An early proof of concept used HSC70‐binding motifs fused to the polyglutamine‐binding peptide QBP1, which selectively promoted the degradation of mutant huntingtin and improved disease‐associated phenotypes in an R6/2 Huntington disease mouse model [115]. This strategy was subsequently developed into a more modular peptide‐based system comprising a protein‐binding domain (PBD), a CMA‐targeting motif (CTM), and a cell membrane‐penetrating domain (CMPD) [116]. The PBD confers target recognition, the CMPD enables cellular entry, and the CTM recruits HSC70 to direct the bound cargo toward LAMP2A‐mediated lysosomal translocation and degradation. Using this architecture, CMA‐directed degradation was extended to endogenous DAPK1, PSD‐95, and α‐synuclein. Their depletion was dependent on lysosomal activity and the CMA machinery, including LAMP2A. Together, these studies established that CMA substrate recognition can be artificially reprogrammed, allowing proteins that do not naturally contain an accessible CMA‐targeting motif to be redirected to the lysosomal degradation pathway.

More recent approaches have expanded both degrader architecture and target scope. Split‐and‐mix CMA‐based degraders (SM‐CMADs) are assembled from self‐assembling peptide modules functionalized with either a KFERQ CMA‐targeting motif or a TBL [117]. By varying the relative proportions of these modules, the platform enables programmable assembly and screening of CMA‐directed degraders. SM‐CMADs incorporating tamoxifen, enzalutamide, cobimetinib, or dasatinib promoted degradation of ERα, AR, MEK1/2, and BCR–ABL, respectively, demonstrating the adaptability of CMA recruitment across multiple oncogenic targets. Antibody–peptide conjugates termed Ab‐CMAs further extended CMA‐based degradation to cell‐surface proteins [118]. By coupling CMA‐targeting peptides to monoclonal antibodies, Ab‐CMAs promoted lysosomal degradation of EGFR, PD‐L1, and HER2 in a manner independent of specific cell‐surface receptors and E3 ligases. These developments broaden the conceptual reach of CMA‐based TPD beyond conventional soluble cytosolic substrates.

CMA‐based degradation therefore provides an alternative to ubiquitin–proteasome and macroautophagy‐based TPD by exploiting the HSC70–LAMP2A axis. It may be particularly useful when target elimination is preferable to occupancy‐based inhibition or when proteasomal recruitment is difficult. However, its direct translocation mechanism imposes an important physical constraint because substrates must remain compatible with unfolding and membrane passage.

## 3 Molecular Design and Determinants of Degradation Efficacy

Degrader efficacy and durability reflect a sequence of coupled events that includes target engagement, recruitment of compatible degradation machinery, formation of a productive complex in the appropriate cellular compartment, pathway commitment, target destruction, and subsequent target recovery. These variables should be evaluated as an integrated process because affinity, linker architecture, or nominal potency alone cannot fully predict cellular degradation [119]. Across TPD modalities, the central design question is therefore not simply whether a degrader binds its target, but whether the target and degrader can complete an entire degradation cycle in the relevant biological context.

### 3.1 Target Engagement and Selection of Degradation Machinery

Target recognition is the first determinant of degradability, but the relevant variable is the molecular state in which the target is presented to the degradation machinery. A ligand can bind with high affinity yet fail to induce degradation if the bound state does not expose a productive surface, if the target is embedded in a protein complex that masks critical interfaces, or if the recruited machinery is absent from the same compartment. For bifunctional degraders, the binding site, binding pose, and exit vector determine how the target surface is oriented after recruitment [11, 39, 120]. Protein conformation, macromolecular interactions, and subcellular localization further alter target accessibility and the geometry available for downstream processing [121, 122]. Thus, a suitable TBL is necessary but not sufficient. The complete degrader must connect a degradable molecular state of the target with a functional disposal pathway in the same biological context.

For ubiquitin‐dependent PROTACs, this compatibility problem extends from the target ligand to the recruited E3 ligase. The target‐binding site and exit vector determine which surface and lysines are presented to the ligase, while recruiter choice determines where and in which cells that presentation can be processed. CRBN and VHL remain widely used because their ligands and complexes are well characterized, but activity depends on the abundance, localization, catalytic competence, and genetic integrity of the complete ligase complex rather than expression of an isolated recruiter. Expanding the E3 repertoire can therefore solve specific biological constraints. DCAF16 provides a nuclear‐restricted recruitment opportunity, whereas DCAF1‐based PROTACs retained degradation activity in models resistant to CRBN‐ or VHL‐dependent routes [123, 124]. Tissue‐enriched machinery can add a further selectivity layer. DT2216 exploits limited VHL availability in platelets to reduce platelet toxicity associated with BCL‐ xL depletion, while KLHL41 recruitment provides a newer strategy for muscle‐restricted degradation [125, 126]. These examples show why recruiter selection should be treated as a biological design variable rather than as an interchangeable chemical handle.

Molecular glues illustrate a different form of machinery compatibility because their activity is governed by formation of a new composite recognition surface rather than by two independently optimized ligands joined by a linker. DCAF15–RBM39 and DDB1–cyclin K systems show that relatively weak binary interactions can become productive when the compound complements a favorable protein–protein interface [51, 52]. Systematic studies of cyclin K glues further showed that solvent‐exposed substituents can tune DDB1 engagement and degradation independently of classical kinase inhibition, demonstrating that target occupancy and glue activity can be separated [127]. Intramolecular bivalent glues extend this principle by engaging adjacent regions of the target in cis, reshaping its surface and promoting E3 recruitment [58]. For molecular glues, surface complementarity, induced target conformation, and latent target–ligase compatibility are therefore more informative than binary affinity alone.

Direct proteasome recruitment and HyT impose still different requirements. CAP‐TACs recruit proteins directly to proteasome cap components and can bypass the conventional need for an E3 recruiter [33]. Earlier direct‐recruitment studies likewise established proteasomal subunits as chemically accessible anchors for TPD [30]. However, proximity to the proteasome is not itself sufficient because the substrate must still be captured, unfolded, and translocated into the catalytic core. HyT instead creates an artificial protein‐quality‐control signal. Its outcome depends on hydrophobic‐tag chemistry, linker architecture, target context, chaperone recognition, and the proteolytic pathway that interprets the induced hydrophobic surface [62, 63, 128, 129]. These strategies reinforce the same general principle: induced proximity is useful only when it connects the target to a downstream processing step that can physically complete degradation.

For extracellular and membrane proteins, the dominant compatibility constraint shifts from cytosolic E3 recruitment to productive trafficking. LYTACs require an accessible target epitope and an internalizing receptor capable of endosomal sorting and lysosomal delivery [14, 28]. A genome‐wide analysis of CI‐M6PR‐based LYTACs showed that receptor occupancy, retromer‐dependent recycling, and lysosomal maturation independently regulate degradation [28]. Endogenous M6P‐modified ligands can occupy CI‐M6PR, while retromer‐mediated recycling can return receptor complexes toward the cell surface instead of the lysosome. Total receptor abundance is therefore an incomplete predictor of activity. Membrane E3‐recruiting platforms such as AbTACs, PROTABs, and REULRs add a geometric requirement because both the target ectodomain and the transmembrane E3 must be accessible in an orientation compatible with internalization and processing [67, 68, 69, 83, 84]. Thus, extracellular degraders should not be viewed simply as binding reagents attached to lysosomal shuttles. Their activity depends on how surface recognition is converted into directional intracellular trafficking.

Autophagy‐directed systems impose another physical constraint because the target or cargo must be captured by an autophagic structure and delivered through a functional lysosomal pathway. ATTECs require simultaneous engagement of the target and LC3, so target affinity, LC3 recognition, linker geometry, cargo size, and the ability of the resulting complex to be sequestered can all affect degradation [22, 90]. AUTACs use a degradation tag to promote selective autophagy, whereas AUTOTACs recruit and activate p62/SQSTM1 to drive cargo clustering and autophagic capture [87, 103]. CMA is more restrictive. HSC70‐mediated recognition must be followed by LAMP2A‐dependent docking, substrate unfolding, and translocation across the lysosomal membrane [86, 110, 111, 112, 113]. A strongly bound substrate can therefore remain poorly compatible with classical CMA if it cannot unfold or traverse the translocation machinery. Ab‐CMA should accordingly be distinguished as a noncanonical extension of CMA‐associated targeting to cell‐surface proteins rather than classical CMA of soluble cytosolic substrates [118]. Collectively, these modalities show that selection of degradation machinery is ultimately a decision about which cellular pathway can physically process the target in its native location and molecular state.

### 3.2 Productive Complex Formation and Degradation Kinetics

Once target and machinery compatibility are established, degradation depends on whether the induced assembly adopts a productive geometry. This point is best defined for PROTACs. Linker length, attachment position, exit vector, flexibility, polarity, and stereochemistry determine the conformational ensemble available to the target–degrader–E3 complex. The MZ1–VHL–BRD4^BD2^ structure showed that degrader‐induced protein–protein contacts can stabilize a cooperative ternary complex and contribute to degradation selectivity [120]. Related p38 PROTACs demonstrated that changing linker attachment and length can alter isoform selectivity even when the same target ligand and VHL recruiter are retained [130]. More recent SMARCA2 studies further showed that degraders with similar static interfaces can display different degradation efficiencies because their dynamic ensembles sample productive conformations with different frequencies [121]. Ternary‐complex affinity and cooperativity are therefore useful descriptors, but neither is a universal predictor of degradation. What matters is whether the ensemble places the substrate in a geometry that can proceed to the next catalytic step.

For ubiquitin‐dependent degradation, that commitment step is ubiquitin transfer rather than ternary‐complex formation itself. Detectable ternary complexes can form without efficient ubiquitination, as shown in the p38 system [130]. Modeling of complete CRL2^VHL^ assemblies showed that more active SMARCA2 degraders positioned a greater density of accessible target lysines near E2‐loaded ubiquitin [121]. Cryo‐electron microscopy of CRL2^VHL^–MZ1–BRD4^BD2^ provided direct structural support by positioning BRD4 toward the E2 active site and identifying a lysine‐rich surface compatible with ubiquitin transfer [39]. Even successful ubiquitination does not guarantee destruction. Ubiquitin‐chain architecture can influence degradation efficiency. For example, TRIP12 cooperates with CRL2^VHL^ to generate K29/K48‐branched ubiquitin chains that accelerate MZ1‐induced BRD4 degradation [131]. The proteasome must then recognize the substrate, engage an initiation region, unfold the protein, and translocate it into the 20S core [30, 39]. Direct proteasome recruiters bypass the initial E3‐dependent delivery step, but they remain subject to substrate‐capture and processing requirements [35]. The productive state is therefore a sequence of geometrically and kinetically coupled events rather than a single stable complex.

Concentration and residence time further shape this sequence. At excessive PROTAC concentrations, separate target–degrader and E3–degrader binary complexes can compete with productive ternary assembly, producing the characteristic hook effect [27]. Positive cooperativity can broaden the concentration range over which productive ternary complexes predominate, although the magnitude of this effect remains system dependent. Residence time can also matter when it provides sufficient opportunity for ubiquitin transfer. MZ1 formed relatively long‐lived VHL complexes with BRD2BD2 and BRD4BD2, and longer dissociative half‐lives correlated with faster initial degradation than observed for BRD3 in that system [132]. However, residence time should not be interpreted in isolation because a long‐lived but geometrically unproductive complex may still degrade poorly. Real‐time measurements further show that degraders with similar endpoint DC_50_ or *D*_max_ values can differ substantially in degradation onset, initial rate, time to maximum depletion, and recovery after compound removal [133]. DC_50_ and *D*_max_ at a single time point therefore compress several mechanistically distinct processes into two numbers. Target resynthesis must also be considered because observed protein abundance reflects the balance between induced destruction and continuing synthesis.

The meaning of a productive complex changes in molecular‐glue systems. Here, the key event is formation of an induced protein–protein interface that positions a neosubstrate for ubiquitination. Small changes in the gluing moiety can reorganize this interface and markedly alter degradation without proportionally changing binary target affinity [51, 52, 58, 127]. This explains why optimization based only on inhibitor potency can miss active molecular glues. The relevant structure–activity relationship includes the ternary surface itself: compound contacts with both proteins, the conformation induced in the target, and the orientation of the recruited substrate relative to the ligase. In this setting, weak binary binding can still support potent degradation if the induced interface is sufficiently complementary and catalytically productive.

For LYTACs and related extracellular degraders, the productive state is not a stable ternary complex but a trafficking cycle. Target capture must be followed by receptor engagement, internalization, endosomal sorting, and delivery to a degradative lysosomal compartment while competing with receptor recycling [28]. This distinction explains why stronger surface binding does not necessarily produce greater degradation. Valency and spatial organization add another design layer. Multivalent aptamer‐based LYTACs (AptLYTACs) showed that trivalent or pentavalent target‐binding architectures could outperform a monovalent construct, whereas further increasing valency reduced degradation despite similar apparent cell binding, indicating that excessive multivalency can impair productive uptake [134]. Tetrahedral DNA LYTACs further demonstrate that nanoscale arrangement and multivalent receptor engagement can enhance membrane‐protein degradation relative to matched monovalent designs [135]. Membrane E3 degraders add target–E3 geometry and ubiquitination to this trafficking problem. Productive design therefore requires optimization of both the extracellular assembly and what happens to that assembly after internalization.

Autophagy‐directed degraders require a different form of productive processing. Cargo must be captured by a growing autophagic structure and successfully pass through autophagosome maturation and lysosomal fusion. For ATTECs, increased LC3 binding alone cannot establish productive degradation because the target–LC3 complex must be positioned at an autophagosomal membrane in a form that can be physically sequestered [22, 90]. This becomes especially important for large cargo such as mitochondria, where successful degradation requires organelle engulfment rather than removal of a single protein. AUTACs illustrate that the chemical degradation tag can tune selective‐autophagy signaling independently of the target ligand [87]. AUTOTACs add a mesoscale assembly step because p62 engagement must promote oligomerization, cargo clustering, and subsequent LC3‐dependent sequestration [103]. Experimental evaluation should therefore include evidence of autophagic flux and terminal lysosomal degradation rather than LC3 accumulation alone, since increased LC3‐II or puncta can also reflect blocked turnover [85]. Across all of these modalities, the common principle is that binding or induced proximity is necessary but not sufficient. Productive degradation requires successful passage through the pathway‐specific commitment step.

### 3.3 Exposure, Selectivity, and Resistance Determinants

The molecular events described above matter only if a chemically intact active species reaches the relevant tissue, cell, and subcellular compartment at a sufficient local concentration. Effective exposure should be distinguished from plasma concentration or total cellular uptake. This issue is particularly important for heterobifunctional degraders in beyond‐Rule‐of‐Five chemical space, where molecular weight, polarity, conformational behavior, solubility, membrane partitioning, and efflux can strongly influence intracellular availability [136]. Linker‐dependent folding can partly shield polar surface area and thereby affect permeability, meaning that linker optimization can simultaneously change pharmacokinetics and ternary‐complex geometry. Molecular glues are often smaller, but permeability and metabolic stability remain compound specific [7, 31]. LYTACs and antibody‐based extracellular degraders avoid passive cytosolic entry but remain constrained by tissue penetration, receptor occupancy, internalization, and endosomal routing [14, 28]. Autophagy‐directed degraders must reach the relevant intracellular compartment and gain access to both the target cargo and the required autophagic machinery.

Selectivity is likewise generated by successive biological filters rather than by target binding alone. PROTAC degradation selectivity can diverge from binding selectivity because only a subset of bound proteins may form a productive target–degrader–E3 geometry [27]. Recruiter distribution adds another filter. DT2216 illustrates how low VHL availability in platelets can reduce on‐target platelet toxicity while retaining BCL‐xL degradation in VHL‐competent tumor cells [126]. Molecular‐glue selectivity depends on complementarity across the ligase–compound–neosubstrate interface, and CRBN engagement alone does not define the full neosubstrate spectrum [31, 54]. Endocytosis‐dependent degraders replace E3 availability with receptor availability and trafficking. GalNAc LYTACs exploit liver‐enriched ASGPR, whereas CI‐M6PR‐based systems have broader cellular distribution [17]. Autophagy‐directed strategies add cargo state and pathway competence as further filters. These examples emphasize that selectivity should be demonstrated at the level of degradation and downstream biology rather than inferred from the nominal targeting ligand or recruiter.

Durability and resistance arise from the same multistep architecture. Target depletion can persist after free drug concentrations fall because the pharmacodynamic state is governed by both degradation and target resynthesis. RIPK2 PROTACs produced pharmacodynamic effects in rats after compound concentrations became undetectable, while the duration of depletion remained linked to the synthesis rate of RIPK2 [137]. Chronic exposure can select defects in the degradation machinery. Resistance to VHL‐ and CRBN‐recruiting PROTACs has been associated with alterations in the corresponding E3 ligase complexes, while increased MDR1 can lower intracellular PROTAC accumulation and produce intrinsic or acquired resistance [138, 139, 140]. Molecular glues share related liabilities. CRBN mutation, copy loss, structural variation, altered splicing, or mutations at the neosubstrate interface can prevent productive glue‐induced degradation [141, 142]. Switching to an alternative ligase such as DCAF1 can bypass one disabled route, but it replaces rather than removes machinery dependence [124]. HyT may similarly avoid dependence on a specific recruited E3 while remaining dependent on chaperone and protein‐quality‐control pathways.

For lysosomal and autophagic degraders, acquired clinical resistance is less well established, but the relevant checkpoints are already evident. Reduced receptor availability, enhanced recycling, impaired endosomal maturation, or lysosomal dysfunction can lower LYTAC flux. Membrane E3 degraders additionally depend on expression and catalytic integrity of the recruited transmembrane ligase. ATTECs, AUTACs, and AUTOTACs require intact cargo recognition, autophagosome formation, and lysosomal flux, whereas classical CMA requires functional HSC70–LAMP2A machinery and a substrate compatible with unfolding and translocation. TPD candidates should therefore be evaluated using matched measurements of active exposure, machinery abundance and function, pathway‐specific commitment, degradation depth and rate, target recovery, and downstream response. The central question is not whether a degrader lowers protein abundance at one endpoint, but whether the complete degradation cycle operates efficiently, selectively, and durably at pharmacologically achievable exposure.

## 4 Degrader Delivery and Spatiotemporal Control

Activity observed in cellular assays does not necessarily translate into therapeutic efficacy in vivo. A degrader must reach the diseased tissue, access the relevant cells, and remain available in an active form. Delivery, cellular entry, and release therefore connect molecular design with therapeutic application.

### 4.1 Non‐Nanoparticle Delivery and Activation

For accessible tissues, local administration can place a degrader near its intended site of action. In a mouse model of androgenetic alopecia, the topically applied AR degrader C6 accumulated in the skin, induced AR degradation, and promoted hair growth [143]. Another study incorporated an AR PROTAC into dissolving microneedles to deliver it through the skin to hair follicles [144]. Local administration has also been investigated for LYTACs. Following intravitreal injection, a bispecific aptamer‐based LYTAC induced VEGF degradation and reduced pathological retinal neovascularization and vascular leakage in mouse models [145]. The choice of local delivery method therefore depends on the location of the lesion and the barriers surrounding it.

For lesions requiring systemic administration, molecular conjugation can influence which cells encounter a degrader. A ROR1 antibody–PROTAC conjugate induced BRD4 degradation after binding to and entering ROR1‐expressing cells. In the mouse models studied, it also showed improved pharmacokinetics and antitumor activity compared with the unconjugated PROTAC [25]. Conjugation has likewise been used to direct lysosomal degraders toward selected cells. Folate receptor‐targeting chimeras degraded cell surface and extracellular proteins in tumor models [74]. A transferrin receptor‐binding peptide was incorporated into a peptide‐based lysosomal degrader, termed Pep‐TAC, that showed PD‐L1 degradation activity in a mouse brain tumor model [146]. These designs use different recognition components to influence where degradation occurs, without relying on a nanoparticle carrier.

For autophagy‐directed degraders acting on intracellular cargo, conjugation can also facilitate cellular entry. An autophagy‐targeting nanobody chimera linked to a cyclic cell‐penetrating peptide entered cells and induced HE4 degradation [147]. In a separate study, intravenously administered Tat–SIRT5–CTM crossed the blood–brain barrier in mice and induced lysosomal degradation of SIRT5 [148]. These studies address cellular uptake and access to brain tissue, respectively.

Conditional activation provides control after a degrader has been administered. Dual‐action‐only PROTACs were designed to require both hypoxia and cathepsin‐L for activation [26]. A related strategy has been applied to LYTACs: conjugating a glutathione‐responsive caged glycan to an anti‐PD‐L1 antibody produced a pro‐LYTAC with condition‐dependent PD‐L1 degradation [149]. Conditional activation can therefore regulate degrader activity in both proteasomal and lysosomal systems.

Local administration, molecular conjugation, and conditional activation address different stages of delivery. Nanocarriers provide a further means of combining degrader loading with control over tissue distribution and release. This creates opportunities to improve degrader exposure in tissues where a single delivery strategy may be insufficient.

### 4.2 Nanotechnology‐Enabled TPD

The intracellular trafficking requirements differ based on the mechanism of action of each TPD technology. For PROTACs, which require the degradation of intracellular proteins, their function depends on the UPS in the cytoplasm. Therefore, the design of nanocarriers for PROTACs must prioritize mechanisms that ensure efficient escape from endosomes. Several strategies have been developed to address this challenge. pH‐responsive systems exploit the “proton sponge effect” of polymers like polyethyleneimine, leading to osmotic swelling and rupture of acidic endosomes [150]. Acid‐labile chemical bond strategies use cleavable linkers that break in acidic environments, inducing membrane disruption through chemical or mechanical stress. Membrane fusion‐based approaches utilize fusogenic materials that merge with endosomal or lysosomal membranes, allowing direct cytoplasmic delivery [151]. In contrast, nano‐LYTACs direct extracellular or cell‐surface targets to lysosomes through receptor‐mediated internalization. Their design should promote cell‐selective target uptake and subsequent lysosomal trafficking. Endosomal escape into the cytosol is generally unnecessary for this degradation route. AUTAC acts on intracellular targets and requires entering the cytoplasm or other related intracellular sub‐regions before inducing K63‐linked ubiquitination and autophagy‐mediated degradation. The design of nano‐AUTAC should achieve controlled release and endosomal escape of the active degrader to enable access to intracellular targets. These differences guide nanoplatform design for each TPD modality. Table 1 summarizes their target locations, degradation pathways, and corresponding delivery and molecular design considerations.

**TABLE 1:** Nanoplatform design considerations for different TPD modalities.

TPD modality	Target location	Degradation pathway	Representative recruited machinery	Requirement for endosomal escape	Delivery strategy	Molecular design considerations	References
Nano‐PROTAC	Intracellular proteins (cytosolic or nuclear)	Ubiquitin–proteasome system	E3 ubiquitin ligases (e.g., CRBN, VHL)	YES. Endosomal escape is typically required after endocytosis to release PROTACs into the cytoplasm.	Nanocarriers (polymeric nanoparticles, liposomes, or inorganic nanoparticles) improve solubility, stability, and tumor targeting.	Target and E3 ligand selection, together with linker geometry, governs productive ternary‐complex formation.	[2, 6, 9, 152]
Nano‐LYTAC	Extracellular proteins or membrane proteins	Endocytosis–lysosome degradation pathway	Lysosome‐targeting receptors such as CI‐M6PR or ASGPR	NO. LYTAC intentionally utilizes receptor‐mediated endocytosis followed by lysosomal degradation.	Nanocarriers enhance circulation time and improve receptor‐mediated uptake.	Target‐binding specificity and the valency and spacing of receptor‐binding ligands influence internalization.	[14, 17, 153, 154]
Nano‐AUTAC	Intracellular proteins, protein aggregates, and damaged organelles	Autophagy–lysosome pathway	Autophagy machinery (e.g., K63‐linked ubiquitination, p62/SQSTM1, LC3)	YES. Required for nanocarrier‐mediated delivery to enable cytosolic access and AUTAC‐induced K63‐linked ubiquitination‐mediated autophagy.	Nanoplatforms can facilitate AUTAC delivery and enable controlled or stimulus‐responsive release.	Target‐binding moieties and guanine‐derived autophagy tags should retain their functionality after delivery.	[87, 155]

#### 4.2.1 Nanomaterial Platforms

Nanomaterials possess characteristics such as adjustable size, easily modifiable surface, strong compatibility in load, and controllable release process, which enables them to support TPD from multiple aspects. Besides simply encapsulating degradation agents, nanomaterials can also improve the formulation and systemic exposure of poorly soluble degradation agents, enhance cellular uptake, regulate intracellular transport, and achieve stimulus‐responsive release. Currently, the main nanomaterials used for TPD include lipid‐based carriers, biomimetic vesicles, polymer nanomaterials, inorganic nanoparticles, protein nanosystems, and DNA‐based nanoplatforms. As shown in Figure 3, these materials can not only act as nanocarriers to promote degradation agent delivery and release, but also serve as integrated nanosystems that directly participate in molecular recognition and intracellular transport processes.

**FIGURE 3:**
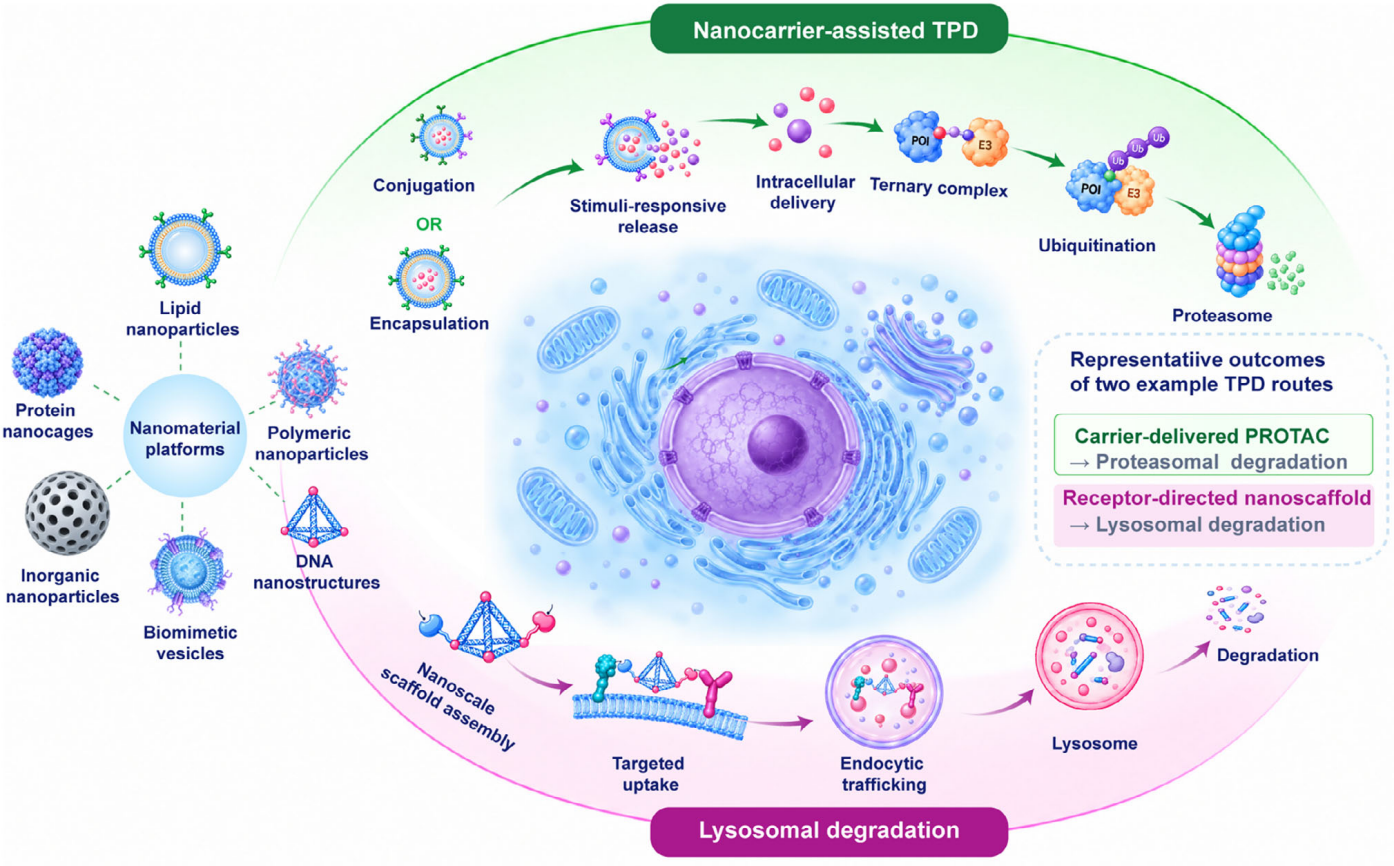
Nanomaterial‐enabled strategies for targeted protein degradation. Representative nanomaterial platforms include lipid nanoparticles, polymeric nanoparticles, protein nanocages, inorganic nanoparticles, biomimetic vesicles, and DNA nanostructures. The upper pathway illustrates nanocarrier‐assisted delivery through degrader encapsulation or conjugation, followed by stimulus‐responsive release, intracellular delivery, E3 ligase recruitment, target ubiquitination, and proteasomal degradation. The lower pathway illustrates nanoscale scaffold assembly, targeted cellular uptake, endocytic trafficking, and subsequent lysosomal degradation.

Lipid‐based nanocarriers mainly include liposomes and lipid nanoparticles (LNPs). Liposomes can simultaneously encapsulate hydrophilic and hydrophobic compounds and enter the endocytic transport pathway after cell uptake [156, 157]. These characteristics are particularly suitable for PROTACs, as their large molecular size and physicochemical properties may limit water solubility and membrane permeability [158, 159]. Therefore, lipid formulations can improve the stability of degraders and tissue exposure [156, 157, 160]. LNPs can further deliver small molecules, peptides, and nucleic acid payloads [161, 162]. Ionizable lipids become protonated in acidic endosomes, enhancing membrane interactions and promoting cytosolic payload release [163]. This process is particularly important for degraders that must access intracellular target proteins and degradation machinery. Thus, the role of the lipid platform is not only to increase particle uptake, but more importantly, to increase the effective exposure of pharmacologically active degraders in the cell.

Bionic vesicles mainly include exosomes, red blood cell membrane vesicles, and bacterial outer membrane vesicles (OMVs). Exosomes retain the membrane components of the source cell and have good biocompatibility, and can transport various cargos such as proteins, lipids, and nucleic acids [164, 165]. These properties are conducive to delivering degradation agents with poor cell permeability or nucleic acid‐encoded degradation systems [166]. Red blood cell membrane vesicles retain membrane proteins such as CD47, which can reduce macrophage clearance and prolong circulation time [167, 168], and their surfaces can be further functionalized to change tissue distribution [169, 170]. Hybrid systems derived from red blood cell membranes have also been used for lysosome–TPD [171]. OMVs have strong cell uptake and cargo delivery capabilities [172, 173, 174, 175], but their own immunological activity needs to be distinguished from protein degradation‐dependent therapeutic effects.

Polymeric nanoparticles and polymeric micelles provide substantial flexibility in molecular architecture and surface chemistry and can be constructed through nanoprecipitation, self‐assembly, or covalent conjugation [176]. Amphiphilic block copolymers can self‐assemble into structures containing a hydrophobic core and a hydrophilic shell, thereby improving the aqueous dispersion of hydrophobic cargos and increasing their stability in circulation [177, 178]. For PROTACs, these features can help address formulation limitations associated with relatively large and hydrophobic molecules. More importantly, polymeric systems can incorporate pH‐, redox‐, or enzyme‐responsive units that trigger particle disassembly or cargo release under defined extracellular or intracellular conditions [177, 178, 179, 180]. Such multistep responsiveness can convert particle accumulation at a disease site into the local availability of an active degrader, making polymeric materials particularly attractive for the spatiotemporal control of nano‐PROTACs. Their structural flexibility also supports barrier‐oriented formulations, such as PROTAC‐loaded PLGA nanoparticles conjugated with CA19‐9 antibodies that enhance intestinal epithelial transport. However, nanoparticle internalization alone does not establish successful TPD. The degrader must be released in a pharmacologically active form and reach sufficient intracellular concentrations to engage both the target and the relevant degradation machinery. Thus, for polymer‐based TPD, release kinetics and productive intracellular exposure are more informative than particle uptake alone.

Inorganic nanoparticles mainly include mesoporous silica, gold nanoparticles, and upconversion nanomaterials. Mesoporous silica has a large specific surface area, tunable pore size, and versatile surface chemistry, enabling efficient drug loading and responsive release [181, 182]. Upconversion nanoparticles can convert near‐infrared light into higher‐energy light. In a representative PROTAC delivery system, this property was exploited to trigger photosensitizer‐mediated reactive oxygen species generation, promoting endosomal escape and cytosolic release of dBET6 for enhanced BRD4 degradation [152]. Gold‐based nanosystems have been investigated for membrane fusion and peptide PROTAC delivery, illustrating their potential to support intracellular transport and TPD [183, 184]. Thus, these materials can function both as delivery carriers and as structural components of nanoscale degradation systems.

Protein‐based nanomaterials mainly include ferritin, albumin, and related self‐assembling protein scaffolds. Ferritin forms well‐defined nanocages, and H‐ferritin‐based nanocages can undergo TfR1‐mediated cellular internalization [185, 186]. Its internal cavity can accommodate therapeutic cargo, whereas its outer surface can support multivalent display of functional ligands. Ferritin can therefore serve both as a delivery carrier and as an active scaffold that promotes receptor‐mediated lysosomal degradation. Recently reported Ferritac and ferritin‐based LYTAC platforms exemplify this mechanism [153, 187]. Albumin has high plasma stability and prolonged systemic exposure [188]. Albumin‐binding or cleavable PROTAC designs can exploit these properties to improve degrader pharmacokinetics, followed by release of the active molecule [189]. Thus, protein‐based nanoplatforms are not merely passive carriers, as their receptor interactions, circulation behavior, and structural features may also influence the tissue distribution and cellular uptake of TPD agents.

DNA and other nucleic acid nanostructures exhibit a high degree of spatial programmability. Through Watson–Crick base pairing, DNA can be assembled into tetrahedra, frameworks, and other structurally defined nanosystems [190, 191]. Their major advantage in TPD lies not only in cargo delivery but also in the precise control of the number, spacing, and spatial orientation of functional ligands. DNA frameworks can simultaneously display TBLs and E3 ligase ligands, thereby organizing degradation components at the nanoscale [192]. Similar principles also apply to DNA‐based LYTACs, in which ligand valency and spatial geometry can influence receptor engagement, internalization, and subsequent lysosomal trafficking [135, 193, 194]. DNA nanostructures can therefore function both as delivery carriers and as geometric templates that directly participate in the degradation process. Their further development still requires improvements in nuclease stability, folding consistency, strand purity, and potential immune recognition.

Overall, different nanomaterials perform distinct pharmacological functions in TPD. The major characteristics, preparation strategies, TPD‐related functions, and translational considerations of these nanomaterial platforms are summarized in Table 2. These material properties can increase the effective exposure of degradation‐active molecules or functional components in the appropriate cells and subcellular compartments, thereby maximizing TPD activity. On this basis, the following sections further discuss how nano‐PROTACs, nano‐LYTACs, and nano‐enabled autophagy‐directed degradation systems use these platforms to meet their distinct delivery and spatiotemporal control requirements.

**TABLE 2:** Nanomaterial platforms for targeted protein degradation.

Material class	Representative preparation methods	Representative construction	Key delivery advantages	Relevant TPD modalities	Safety considerations	Pharmacological performance	Pharmaceutical development considerations	Principal translational limitations	References
Liposomes and lipid nanoparticles	Thin‐film hydration, ethanol injection, or microfluidic mixing; lipid formulation of hydrophobic or peptide‐based degrader cargo	Liposomal or LNP formulation of small‐molecule or peptide degraders, with optional surface functionalization	Improve formulation of poorly soluble degraders and facilitate intracellular delivery of peptide cargo	PROTACs, peptide PROTACs, and CMA‐directed systems	Selected formulations show favorable preclinical tolerability, although safety remains formulation dependent.	Can enhance cellular uptake and in vivo delivery while retaining degradation activity	Lipid composition, drug loading, formulation stability, and release behavior require optimization	Particle uptake does not necessarily ensure sufficient release of the active degrader.	[159, 195, 196, 197]
Polymeric nanoparticles and micelles	Nanoprecipitation; self‐assembly of amphiphilic block copolymers; covalent polymer‐degrader conjugation; pH‐, redox‐, or enzyme‐responsive polymer assembly	Polymeric encapsulation, covalent degrader conjugation, and stimulus‐responsive self‐assembly	Enable controlled or stimulus‐responsive release and improve intracellular or barrier‐crossing delivery	Nano‐PROTACs and nano‐ATTECs	Several polymeric systems show favorable tolerability in preclinical models.	Stimulus responsiveness and release kinetics can modulate intracellular exposure, degradation efficiency, and therapeutic activity.	Particle size, loading or conjugation efficiency, and release kinetics are key formulation parameters	Incomplete or slow release may limit productive intracellular degrader exposure.	[9, 176, 198, 199, 200]
Protein‐based carriers and nanocages	Recombinant expression of ferritin or other protein scaffolds; SpyTag‐SpyCatcher or chemical conjugation; albumin‐assisted coassembly	Ferritin or H‐ferritin nanocages, SpyTag‐SpyCatcher assembly, and covalent protein conjugation	Support multivalent presentation and receptor‐mediated internalization	Ferritac, ferritin‐based LYTACs, and ferritin‐conjugated PROTACs	Ferritin‐based degraders have shown favorable tolerability in preclinical studies.	Can enhance receptor‐mediated uptake, tumor delivery, target degradation, and pharmacokinetic behavior	Scaffold integrity, conjugation strategy, ligand accessibility, and payload release require control	The protein scaffold may itself participate in targeting and internalization, linking carrier and degradation effects.	[153, 187, 201]
Inorganic nanoparticles	Gold‐thiol or Au(I)‐thiol assembly; synthesis of gold nanoparticles or nanocubes; mesoporous silica coating of upconversion nanoparticles	Au(I)‐thiol assemblies, gold nanoparticles, upconversion nanoparticle‐based nanocages, and catalyst‐containing nanosystems	Enable multivalent presentation, photoactivation, and bioorthogonal activation	Metal‐assisted degradation systems, nano‐LYTACs, photoactivatable nano‐PROTACs, and bioorthogonal PROTACs	Clearable inorganic systems have shown favorable preclinical tolerability, while safety remains material dependent.	Enable externally triggered or catalyst‐dependent spatiotemporal control of degradation	Inorganic cores, surface functionalization, and activation or catalytic components must be coordinately controlled	Dependence on external stimuli or catalyst codelivery increases multicomponent and spatial‐control requirements.	[202, 203, 204, 205]
DNA nanostructures	Sequence‐programmed annealing of DNA tetrahedra, frameworks, modular assemblies, or dendronized DNA chimeras	DNA tetrahedra, framework structures, programmable nanoassemblies, and DNA origami	Allow precise control of ligand spacing, valency, stoichiometry, and spatial organization	DNA‐based degraders, DNA‐LYTACs, and multivalent lysosomal degraders	Tumor‐responsive and tissue‐selective designs can reduce nontarget activity.	Ligand geometry and multivalency can enhance target engagement, cellular uptake, and degradation efficiency.	DNA stability, ligand stoichiometry, and assembly fidelity require precise control	In vivo stability and reproducible construction of complex DNA architectures remain important development challenges.	[135, 192, 193, 194]
Membrane‐ and vesicle‐derived systems	Membrane extrusion or fusion for biomimetic coatings; engineering or isolation of exosomes, nanovesicles, or bacterial outer‐membrane vesicles	Engineered outer‐membrane vesicles, nanovesicles, extracellular vesicles, and exosomes	Exploit endogenous membrane components for targeting, receptor engagement, and tissue delivery	OMV‐LYTACs, nanovesicles‐based lysosome‐targeting protein degradation platform (NV‐TACs), extracellular‐protein degradation, and autophagy‐directed delivery	Safety depends on vesicle origin; bacterial OMVs can exert intrinsic immunostimulatory activity, whereas selected engineered nanovesicles show favorable preclinical tolerability.	Can improve tumor targeting, receptor‐mediated internalization, extracellular target clearance, or delivery across biological barriers	Vesicle source, functional components, cargo incorporation, and manufacturing consistency require control	Intrinsic vesicle bioactivity may overlap with the effects of targeted protein degradation and complicate attribution of efficacy.	[206, 207, 208, 209]

#### 4.2.2 Nano‐PROTAC

For nano‐PROTACs, cellular uptake is only one step toward target degradation. An active degrader must also become available where it can engage both its target protein and the recruited E3 ligase. This distinction is relevant to formulations as different as nanoliposomes carrying EGFR‐ and BRD4‐directed degraders, mixed‐shell micelles carrying dBET6 and CXCL9, and gold nanoclusters displaying HER2‐binding and CRBN‐recruiting groups [159, 210, 211]. Each system has a different therapeutic purpose, but particle uptake alone cannot establish that its PROTAC component has reached a productive intracellular site. Intracellular release and target–protein depletion therefore remain necessary measures of degrader activity.

##### 4.2.2.1 Self‐Assembly and Carrier‐Free Nano‐PROTACs

Self‐assembly provides an alternative to conventional nano‐PROTAC formulations in which degraders are loaded into separately prepared carriers. Earlier nano‐PROTAC designs already exploited self‐assembling nanomaterials for controlled degrader delivery. For example, ARV‐771 was encapsulated within redox‐responsive poly(disulfide amide) nanoparticles that disassembled under reducing conditions to promote intracellular degrader release [198].

More recent studies have extended this concept toward carrier‐free formulations. dBET57 and chlorin e6 formed nanoparticles through noncovalent coassembly without additional drug excipients [212]. PROTACs have also been coassembled with cyanine dyes such as IR783 and indocyanine green to generate supramolecular nanoparticles with PROTAC contents of up to approximately 70 wt% without exogenous carriers [213]. Alternatively, self‐assembly can be encoded directly into the degrader architecture. Amphiphilic EGFR‐targeting PROTACs spontaneously formed stable nanoparticles without external excipients and showed improved aqueous solubility compared with the corresponding free PROTAC [214]. Similarly, incorporation of an alkyl linker and a fatty acid into an aptamer‐derived PROTAC generated a dual‐hydrophobic module that promoted nanomicelle formation, increased cellular internalization by approximately fivefold, and enabled efficient intracellular sclerostin degradation [215].

These studies indicate that hydrophobicity and amphiphilicity, which can otherwise contribute to unfavorable properties of free PROTACs, may be exploited to support nanoscale assembly. However, self‐assembly should not automatically be equated with a completely carrier‐free formulation because stabilizers, targeting coatings, dyes, or other auxiliary components may still be present. For intracellular targets, the assembled system must also disassemble or release a degradation‐competent PROTAC at a site where both the target protein and the recruited E3 ligase are accessible. By reducing dependence on separately manufactured carrier materials, carrier‐free self‐assembling PROTACs may simplify formulation composition and manufacturing requirements and thereby improve their potential for clinical translation.

##### 4.2.2.2 Conditional Activation and Release

Conditional activation and release provide a mechanistic means of separating the requirements for systemic delivery from those for intracellular protein degradation. Free PROTACs must retain sufficient solubility and stability during administration and circulation, reach the appropriate tissue and cell, and subsequently achieve an intracellular concentration that permits productive engagement of both the target protein and the recruited E3 ligase. Nanoformulation can partly decouple these requirements by maintaining the degrader in a protected, solubilized, conjugated, or inactive state during transport and converting it into a degradation‐competent species only after reaching the intended biological environment. The relevant nano‐effect is therefore not simply increased particle uptake. Rather, nanotechnology can redistribute the functions required for PROTAC activity across successive stages of delivery, with the formulation governing systemic transport, tissue distribution, cellular entry, and release, and the liberated degrader retaining responsibility for productive target–E3 ligase engagement.

Endogenous redox conditions provide several chemically distinct mechanisms for this conversion. ARV@PDSA encapsulates ARV‐771 within a poly(disulfide amide) matrix whose disulfide bonds stabilize the polymeric network. Elevated intracellular glutathione reduces these bonds, destabilizing the polymer backbone and causing particle disassembly and ARV‐771 release [198]. Nano‐MP@PSI, a supramolecular gold(I)‐thiol‐peptide complex (Nano‐MP) coated with pH‐responsive polyacryl sulfydryl imidazole (PSI), instead relies on reversible Au(I)‐thiol coordination. Protonation of the PSI coating under acidic conditions promotes cellular uptake, after which intracellular glutathione disrupts the Au–S interactions, leading to nanostructure disassembly and release of the MDMX‐directed peptide degrader [202]. Related systems show that reduction sensitivity can also be combined with cell‐specific delivery. A dual‐targeting nano‐PROTAC termed CREATE, constructed from CRV‐engineered Lewis lung carcinoma cell membranes, pH/GSH‐responsive DS‐PLGA, and the BRD4 degrader dBET6, was designed to target both lung cancer cells and tumor‐associated macrophages. In subcutaneous and orthotopic LLC tumor models, CREATE promoted BRD4 degradation in both cell populations, induced apoptosis, and suppressed tumor growth [216]. A GSH‐responsive nanocapsule carrying SIAIS171142 similarly couples disulfide crosslinker cleavage to BRD4 degrader release while incorporating a glioblastoma‐targeting feature [217]. ROS can provide an alternative endogenous trigger. In BT‐NanoPROTAC, a ROS‐responsive boronic acid‐modified ARV‐771 derivative forms nanoparticles with Poloxamer 188 and releases active ARV‐771 in activated hepatic stellate cells, extending conditional nano‐PROTAC activation beyond oncology [218]. Thus, disease‐associated redox conditions can control polymer disassembly, disruption of coordination interactions, cleavage of crosslinkers, or chemical unmasking of a degrader.

Acidity and enzymatic activity can further divide delivery into sequential steps. In CDK4/6‐directed sequentially responsive nanoparticles, mildly acidic conditions in the tumor microenvironment induce particle transformation into smaller assemblies that improve penetration and cellular uptake. Following internalization, lysosomal cathepsin B cleaves the Gly–Phe–Leu–Gly‐containing linker connecting the polymer to the PROTAC, releasing the active degrader and increasing CDK4/6 depletion [9]. An ALK‐directed platform applies a similar cascade, in which acid‐responsive structural transformation precedes cathepsin B‐mediated release of the ALK degrader and subsequent reductions in ALK and phosphorylated ALK [219]. Enzyme‐responsive activation can also be incorporated directly into the degrader‐containing nanostructure. In SPNpro, an indoleamine 2,3‐dioxygenase (IDO)‐targeting PROTAC peptide is linked to a semiconducting polymer through a cathepsin B‐cleavable peptide. Proteolytic cleavage releases the degrader in CatB‐rich tumor cells, whereas the semiconducting polymer independently generates singlet oxygen under near‐infrared irradiation [220]. In BALB/c mice bearing primary and distant 4T1 tumors, SPNpro promoted IDO degradation and enhanced antitumor immune responses. pH‐responsive materials can additionally modify cellular entry itself. In the nChap platform, protonation of poly(β‐amino ester) in acidic tumor tissue alters the micellar state and surface charge, facilitating cellular uptake and dBET6 release while the same carrier delivers CXCL9 (Figure 4A) [211]. These systems illustrate that acidity or enzyme activity does not merely trigger drug release. It can regulate particle size, surface properties, tumor penetration, cellular uptake, and the final generation of intracellularly available PROTAC.

**FIGURE 4:**
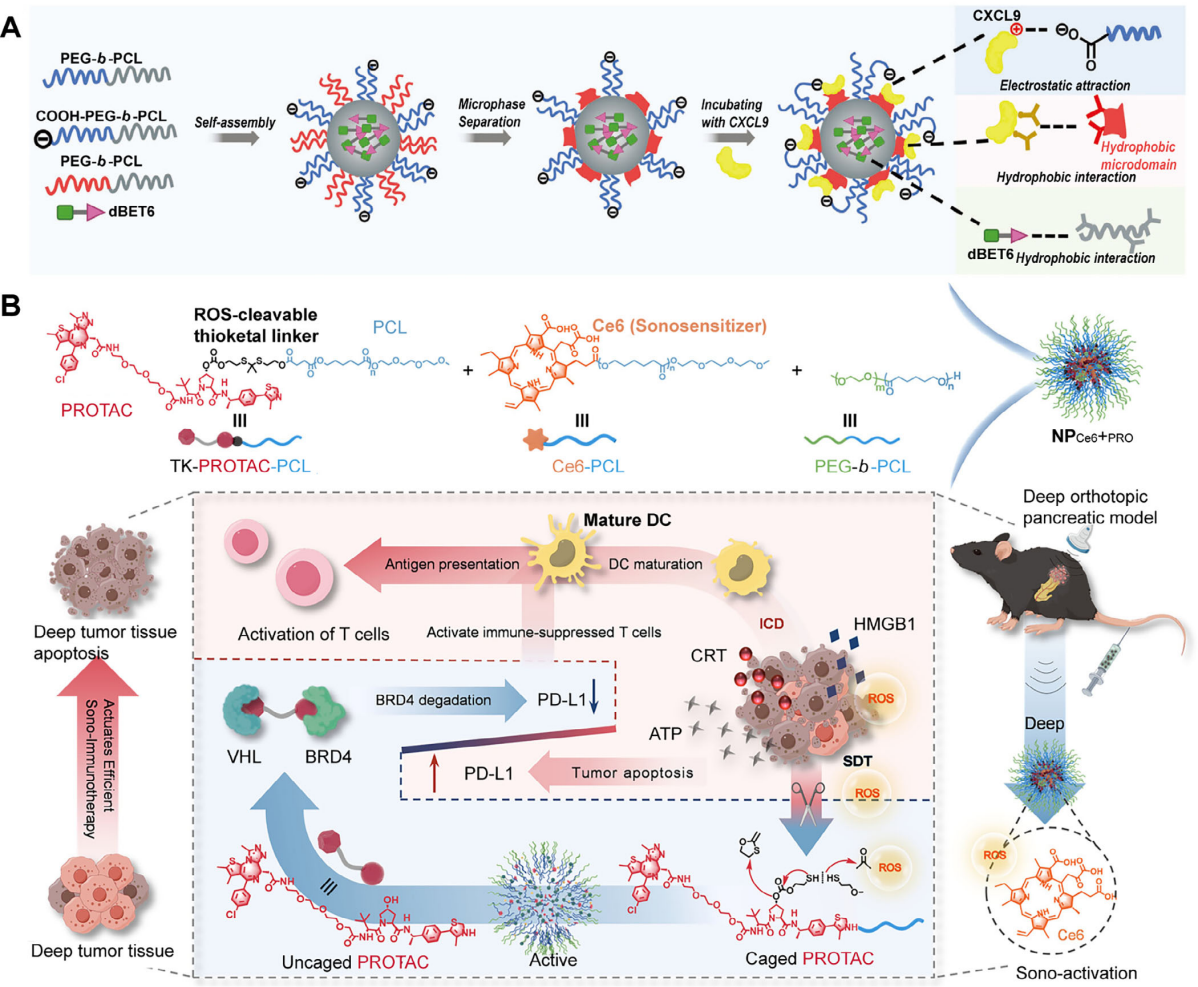
Representative stimuli‐responsive nano‐PROTAC platforms for controlled delivery and protein degradation. (A) Diagram depicting the preparation of pH‐responsive nano‐PROTAC and the mechanism for loading CXCL9 and dBET6 using a nanochaperone. Reproduced from ref. [211] with permission from John Wiley & Sons, copyright 2024. (B) Schematic illustration of ROS‐responsive NP_Ce6+PRO_ formation and its mechanism for protein degradation and tumor therapy. Reproduced from ref. [224] with permission from American Chemical Society, copyright 2024.

External stimuli provide greater temporal control over the same delivery‐to‐degradation sequence. In a self‐assembled near‐infrared‐responsive platform, ARV‐771 is linked to pyropheophorbide a through a thioketal linker. Irradiation activates the photosensitizer and generates singlet oxygen, which cleaves the thioketal linkage and releases ARV‐771 for BRD4 degradation [221]. Upconversion nanomaterials use near‐infrared irradiation through mechanistically different routes. UMSNs@phoBET1 converts 980nm excitation into higher‐energy emission that photolyzes the caging group of phoBET1 and restores an active BRD4 degrader [204]. By contrast, a nanodevice carrying unmodified dBET6 uses upconversion‐mediated activation of Rose Bengal to generate reactive oxygen species that promote endolysosomal escape and cytosolic release of dBET6 [152]. In the latter case, the PROTAC itself is not chemically cleaved or uncaged. Instead, the nano‐effect alters intracellular trafficking, emphasizing that cellular uptake alone cannot establish productive PROTAC delivery. This problem has been addressed more directly by an ionizable nano‐PROTAC in which tertiary amines cooperate with a hydrophobic C16 segment to enhance cellular uptake and promote proton‐sponge‐mediated endosomal escape, thereby increasing cytosolic delivery and Leucine‐rich α‐2‐glycoprotein 1 degradation [222].

Other externally applied energy sources regulate analogous processes in deeper tissues. X‐ray‐responsive RCNprotac contains MZ1 conjugated to polyethylene glycol through a diselenide‐containing linker. Irradiation disrupts the radiation‐sensitive linkage and releases MZ1 for BRD4 degradation [223]. In the ultrasound‐responsive NP_Ce6+PRO_ platform, chlorin e6 generates reactive oxygen species under ultrasound irradiation, which cleave a thioketal linkage and release a BRD4‐directed PROTAC in pancreatic tumor models (Figure 4B) [224]. More complex designs use multiple tumor‐associated cues to address intratumoral heterogeneity rather than relying on a single stimulus. PGDAT@N combines MMP‐2‐responsive dePEGylation, acid‐dependent nanostructure changes, ROS‐cleavable thioketal linkages, and a hypoxia‐responsive ARV‐771 precursor. This design promotes tumor accumulation and penetration while allowing BRD4 degradation in differently oxygenated tumor regions [225]. These examples show that spatial selectivity can arise not only from nanoparticle accumulation, but also from restricting degradation‐competent PROTAC formation to tissues or regions in which the required trigger is present.

Bioorthogonal chemistry extends this principle by generating an active degrader after delivery. In AuPLs, a protected dBET1‐derived pro‐PROTAC and a water‐soluble Pd catalyst are codelivered within gold nanocubes coated with a lauric acid/stearic acid phase‐change layer. Near‐infrared photothermal heating melts the coating and releases both components, allowing Pd‐mediated deprotection and restoration of BRD4‐degrading activity [205]. A catalyst‐free strategy masks the VHL‐binding region of MZ1 with a 4‐(vinyloxy)benzyl carbonate group to generate MZ1‐O. Targeted nanoparticles deliver MZ1‐O systemically, whereas a locally administered tetrazine‐containing microneedle initiates an inverse electron‐demand Diels–Alder reaction followed by bond cleavage and 1,6‐elimination to regenerate active [226]. Nano‐CLIPTACs go further by dividing an ALK‐directed PROTAC into two smaller precursors that are separately encapsulated in cyclic RGD‐modified liposomes [227]. After tumor‐targeted delivery, intracellular bioorthogonal reaction generates the complete functional PROTAC. The study also demonstrated an anti‐hook effect, indicating that controlling where and when the complete degrader is formed can alter the concentration conditions governing productive degradation. Bioorthogonal chemistry can additionally be used at the level of tumor retention. In POLY‐PROTAC nanoparticles, extracellular acidity exposes DBCO groups on pretargeted nanoparticles, enabling click‐mediated retention of azide‐functionalized POLY‐PROTACs at the tumor site. Subsequent intracellular reduction of disulfide bonds releases the active BRD4 degrader [228]. Thus, bioorthogonal reactions can control degrader uncaging, in situ PROTAC assembly, or spatial enrichment before intracellular release.

Nanoformulation can also reshape the tissue selectivity of PROTACs by altering their distribution and restricting where the active degrader becomes available. Nanoparticle size, surface properties, and formulation characteristics influence circulation and tissue exposure, while membrane camouflage or targeting ligands can further bias delivery toward selected tissues or cell populations. Microenvironment‐responsive release and externally controlled activation provide additional layers of spatial restriction by limiting the generation of degradation‐competent PROTAC to defined pathological or treated regions. Thus, tissue selectivity in nano‐PROTAC systems arises from the combined control of biodistribution, cellular delivery, and local degrader activation rather than from nanoparticle accumulation alone.

More broadly, these systems show that nanotechnology reshapes PROTAC behavior through a sequence of mechanistically distinct processes rather than through passive encapsulation alone. Nanoformulation can improve formulation and systemic transport, enhance tissue penetration and cellular uptake, facilitate endosomal escape, and regulate the intracellular availability of active PROTACs through nanostructure disassembly, bond cleavage, uncaging, or in situ synthesis. In this way, part of the physicochemical burden associated with systemic delivery is transferred from the PROTAC molecule to the formulation, whereas the released or regenerated degrader remains responsible for productive target–E3 ligase engagement.

Nanoformulation may also influence the hook effect by controlling the intracellular availability of active PROTACs. Responsive matrices, cleavable linkers, and triggered nanostructure disassembly can delay degrader release during transport. This can reshape the intracellular concentration–time profile of free PROTAC. Excessively rapid release may transiently generate high intracellular concentrations and favor nonproductive binary complexes. In contrast, overly slow release may provide insufficient active degrader for efficient ternary‐complex formation. Nano‐CLIPTACs provide a more direct example. Two PROTAC precursors are delivered separately and form the complete degrader only after an intracellular bioorthogonal reaction. This controlled formation of active PROTAC was associated with a reduced hook effect [227]. Thus, nanomaterials may modulate the hook effect by regulating the rate and timing of intracellular PROTAC release or generation.

Whether these mechanistic advantages translate into meaningful pharmacological benefit ultimately depends on their pharmacokinetic and pharmacodynamic consequences. Prolonged circulation or increased tumor accumulation is pharmacologically meaningful only when it increases the effective exposure of degradation‐competent PROTACs within target tissues and relevant intracellular compartments, thereby producing measurable target depletion. Several studies have shown that nanoformulation can translate improved pharmacokinetics into enhanced protein degradation. In CT26 tumor‐bearing mice, a single dose of a sequentially responsive CDK4/6 nano‐PROTAC produced approximately 2.6‐fold and 3.0‐fold greater depletion of tumor CDK4 and CDK6, respectively, than the corresponding free PROTAC at the reported matched dose [9]. Similarly, an ALK‐targeting nanoplatform linked improved systemic exposure and tumor accumulation to greater reductions in ALK and phosphorylated ALK levels [219]. P‐selectin‐targeted Fi‐dBET6 nanoparticles also substantially altered the in vivo disposition of dBET6, extending its plasma half‐life from approximately 30 min to 3 h and increasing its concentration in tumor tissue. At the same 15 mg/kg dose, Fi‐dBET6 induced marked BRD4 degradation in tumors, whereas free dBET6 did not produce significant target depletion [229]. However, improved pharmacokinetics does not necessarily translate into stronger pharmacodynamic activity. Compared with the solution formulation, liposomal encapsulation of GNE‐01 increased systemic exposure by approximately 80‐fold in mice and 380‐fold in rats, yet this substantial increase in exposure did not result in greater BRM degradation in tumors, possibly because of slow PROTAC release from the liposomes [230]. These findings indicate that prolonged circulation or enhanced tissue accumulation alone is insufficient to ensure improved protein degradation. Rather, active degrader must be released at an appropriate rate and achieve intracellular concentrations that support productive ternary‐complex formation.

##### 4.2.2.3 Applications Beyond Oncology

Nononcological studies apply nano‐PROTAC delivery to barriers other than those encountered in solid tumors. A reactive oxygen species‐activatable formulation was developed to release an ARV‐771‐derived BRD4 degrader in activated hepatic stellate cells and was evaluated in liver fibrosis models [218]. For atherosclerosis, a METTL4 degrader was loaded into PLGA nanoparticles coated with M2 macrophage membranes to enhance delivery to macrophages within atherosclerotic plaques [231]. In choroidal neovascularization, a tetrahedral DNA construct termed dNCL@tFNAs was developed to improve delivery of a nucleolin‐directed degrader. Direct addition of free dNCL to endothelial cells did not produce the target depletion observed with dNCL@tFNAs, whereas proteasome inhibition blocked degradation induced by the DNA construct [232]. These studies connect carrier design to the conditions of the diseased tissue: oxidative stress in activated stellate cells, cellular localization within plaques, and limited access across ocular barriers.

Combination formulations are also being investigated outside oncology. In rheumatoid arthritis models, a self‐assembled nano‐PROTAC paired a COX‐2‐directed degrader with betulinic acid, which contributes biological activity in addition to the PROTAC component [233]. This distinction also matters for the liver fibrosis and atherosclerosis platforms, in which formulation changes may affect which cells receive the degrader, and for dNCL@tFNAs, where a comparison with directly added dNCL links improved delivery to observed nucleolin loss [218, 231, 232]. Collectively, these studies indicate that the value of a nano‐PROTAC depends on the barrier it addresses and the evidence connecting delivery to target degradation. Where another active treatment is incorporated, its contribution to disease improvement must be distinguished from that of protein depletion.

#### 4.2.3 Nano‐LYTACs

##### 4.2.3.1 Multivalent Organization and Receptor Recruitment

Nano‐LYTACs use nanoscale scaffolds to bring target‐binding modules together with components that promote lysosomal trafficking. The scaffold determines how many binding groups are displayed and how they are arranged, which can influence whether target engagement leads to productive internalization [154, 193, 203]. GalNAc‐modified glycopolypeptide nanospheres recruit the ASGPR, whereas gold nanoparticle‐anchored aptamer chimeras combine ABCG2 recognition with recruitment of IGF2R [154, 203]. DNA nanoassemblies offer a programmable alternative in which the positions of target‐ and receptor‐binding aptamers can be adjusted [193]. Across these systems, increased cell‐surface binding or uptake does not, by itself, establish improved degradation. The bound target must subsequently reach a degradative lysosomal compartment.

Programmable DNA structures provide relatively direct evidence that nanoscale organization affects target degradation. Multivalent DNA LYTACs have been assembled from aptamers recognizing both pathogenic membrane proteins and IGF2R, allowing the spatial distribution of their binding modules to be varied [193]. The intelligent modular DNA LYTAC nanodevice extends this approach by controlling the numbers of target‐binding sites and pH‐responsive modules that promote degradation [194]. In a direct comparison, a tetrahedral DNA LYTAC bearing target‐specific aptamers and trivalent GalNAc induced approximately twice the membrane‐protein degradation observed with the corresponding monovalent aptamer LYTAC in the tested hepatocyte system [135]. A separate series of DNA constructs displaying one, three or nine binding units showed enhanced degradation with increasing valency across the receptor pathways examined [234]. These results support a pharmacodynamic benefit from multivalent presentation under defined conditions, although the most productive arrangement depends on the target and the recruited receptor.

Receptor choice also affects where and how a nano‐LYTAC acts. GalNAc‐bearing nanospheres recruit ASGPR, making receptor expression relevant to the cellular context of degradation [154]. An integrin‐directed nanochimera uses multivalent RGD peptides to engage αvβ3 integrin and attached antibodies to bind PD‐L1. In the reported models, the complex underwent internalization and lysosomal degradation, limiting PD‐L1 recycling [235]. IGF2R‐binding aptamers displayed on gold nanoparticles or DNA assemblies provide a different route for target trafficking [193, 203]. Fibroblast‐derived nanovesicles displaying PD‐1 and transferrin have more recently been engineered to bind PD‐L1 and TfR1, respectively [207]. These studies show that the recruited receptor influences both which cells can internalize a construct and whether the bound target enters a productive degradation pathway.

##### 4.2.3.2 Conditional Control and Alternative Architectures

Some nano‐LYTAC systems couple target degradation to local activation or a second therapeutic function. Semiconducting polymer nanoparticles bearing surface‐assembled peptide structures were reported to induce PD‐L1 degradation while supporting photodynamic treatment [236]. In an acidity‐responsive formulation, changes to the nanoparticle surface promoted the activity of a PD‐L1‐directed nano‐LYTAC within the tumor environment, alongside a catalase‐associated treatment component [237]. Sonodynamic nano‐LYTACs extended the target range to IL‐4 receptor on M2 macrophages and combined receptor degradation with ultrasound‐dependent effects on the tumor microenvironment [238]. Self‐assembling CTGF‐directed LYTAC nanoparticles have been constructed with a glutathione‐responsive linkage, while CAIX‐targeting supramolecular assemblies use membrane‐associated CAIX to facilitate PD‐L1 internalization [239, 240]. Because these formulations may also produce reactive oxygen species or other biological effects, their overall therapeutic activity cannot be attributed entirely to protein degradation.

Protein cages and biological vesicles provide further ways to organize target binding and lysosomal trafficking within one construct. Human heavy‐chain ferritin LYTACs display target‐specific affibodies through SpyTag‐SpyCatcher coupling and recruit TfR1 through an engineered ferritin scaffold [153]. The reported constructs degraded HER2, EGFR, and PD‐L1, with evidence for contributions from both TfR1‐dependent internalization and the particle's multivalent properties [153]. PD‐1/transferrin nanovesicles also recruit TfR1, but present their functional proteins within a vesicle membrane rather than on a protein cage [207]. Engineered bacterial outer‐membrane vesicles provide another formulation for TfR1‐associated PD‐L1 degradation and contribute immunostimulatory activity of their own [206]. Thus, target loss observed with these constructs may involve different trafficking routes and accompanying biological effects, even when they recruit the same receptor.

Multivalent nanostructures can also promote lysosomal degradation without the receptor‐recruiting arrangement of a conventional LYTAC. Ferritac displays multiple antibodies on ferritin to crosslink neighboring membrane proteins and induce internalization without requiring recruitment of an additional lysosomal trafficking receptor [187]. Circular multivalent nanobody‐targeting chimeras recruit scavenger receptors and promote clathrin‐ or caveolae‐associated internalization of bound membrane proteins [241]. Aptamer‐functionalized gold nanoparticles termed PANTACs have been reported to induce membrane‐protein degradation through clathrin‐associated uptake independently of a LTR [242]. These approaches broaden the routes by which a nanoscale construct can direct a bound membrane protein toward lysosomes. Their uptake mechanisms remain distinct from those of nano‐LYTACs that explicitly recruit a lysosomal trafficking receptor.

##### 4.2.3.3 Pharmacodynamic and Pharmacokinetic Evidence

Whether a nano‐LYTAC improves on a non‐nano degrader depends on the comparator and the endpoint measured. The tetrahedral DNA study compared target degradation with that induced by a corresponding monovalent aptamer LYTAC, while a controlled‐valency DNA series examined the effect of binding‐unit number within related constructs [135, 234]. Other comparisons address narrower questions. GalNAc nanospheres increased EGFR internalization relative to free cetuximab, but cetuximab alone lacks the receptor‐recruiting component of a complete LYTAC [154]. These results support a role for nanoscale organization in the tested systems, but comparisons with an antibody or an individual binding module do not establish superiority over a complete, structurally matched non‐nano‐LYTAC.

Evidence concerning systemic exposure requires the same distinction. In a ferritin study, the plasma half‐life estimated from fluorescence measurements was 131.8 min for a HER2‐targeting nanocage construct and 9.3 min for its free HER2‐binding module [153]. This supports greater persistence after incorporation of the binding module into ferritin. The free comparator, however, was not a complete non‐nano‐LYTAC. Attachment of certain target binders also reduced the ferritin construct's affinity for TfR1, indicating that improved target binding or longer circulation need not produce stronger receptor‐mediated degradation. Together with the controlled‐valency DNA studies, these findings show why binding, receptor recruitment, systemic exposure and target depletion need to be interpreted as separate properties of a nano‐LYTAC.

##### 4.2.3.4 Applications Beyond Oncology

Lysosomal nanodegraders are being investigated in inflammatory and ocular diseases. A supramolecular assembly recruiting macrophage scavenger receptor A promoted lysosomal degradation of extracellular IL‐17A and reduced inflammation in a mouse model of psoriasis‐like disease [243]. In ocular disease, a bispecific aptamer LYTAC recognizing VEGF and the M6PR provides a non‐nano example of locally administered extracellular‐protein degradation [145]. A nucleic acid‐based LYTAC Plus hydrogel combined VEGFR‐2 degradation with siRNA‐mediated ANG‐2 silencing in models of neovascular age‐related macular degeneration [244]. More recently, DNA nanoflowers comprising multiple aptamer‐based VEGF‐degrading units were used to carry verteporfin for combined degradation and photodynamic treatment [245]. The hydrogel and nanoflower results reflect more than protein degradation alone, while the individual aptamers used as nanoflower controls are not equivalent to a complete free LYTAC.

Related systems have also been studied in neurological and cardiovascular disease. An endocytosis‐triggering polyvalent nanochimera targeting RAGE was evaluated in models of Alzheimer's disease, addressing both delivery to the relevant tissue and removal of a disease‐associated protein [246]. In acute myocardial infarction, a Ly6G‐targeted, red‐blood‐cell‐mimetic liposomal degrader promoted macrophage SIRPα loss and enhanced efferocytosis through a route distinct from conventional receptor‐recruiting LYTACs [171]. Together with the IL‐17A‐directed assembly, these studies extend nanoscale lysosomal degradation beyond tumor‐cell membrane proteins. They also highlight different requirements across diseases: reaching the relevant tissue, engaging the appropriate cell population and demonstrating loss of the intended protein are related, but separately verifiable, outcomes.

Across these applications, the clearest evidence for a benefit from nanoscale design comes from comparisons between closely related constructs with controlled differences in valency or arrangement. Evidence for superior pharmacokinetics over a complete non‐nano‐LYTAC remains less direct, as illustrated by the ferritin comparison with a free binding module. For combination systems, improvements in disease outcomes must also be interpreted alongside the effects of siRNA, photodynamic treatment or other active components.

#### 4.2.4 Autophagy–Lysosomal Pathway Nano‐TPD

##### 4.2.4.1 Nano‐AUTAC

Unlike nano‐PROTACs, nano‐enabled autophagy‐directed degraders are not evaluated primarily by cytosolic release and E3 ligase engagement. Their activity requires the delivered construct to preserve productive cargo recognition and engagement of the relevant autophagy machinery, followed by completion of autophagic flux and lysosomal degradation. Accordingly, enhanced uptake or tumor accumulation is insufficient unless the nanosystem supports the pathway‐specific steps required for cargo sequestration and degradation. Upon entering the cell, the target protein ligand of AUTAC binds to the target protein, and the degradation tag mimics S‐guanylation modification, inducing polyubiquitination of the target protein [87]. However, the poor water solubility and high polarity of AUTAC molecules result in low bioavailability and permeability in vivo, limitations that can be addressed through supramolecular self‐assembly [155]. Supramolecular artificial nanotechnology is an emerging field that integrates supramolecular chemistry with nanotechnology, relying on noncovalent interactions (e.g., hydrogen bonds, van der Waals forces, and electrostatic interactions) to form nanomaterials with specific structures and functions through self‐assembly [247]. This technology can respond dynamically to external stimuli, such as light, electricity, and temperature [248]. As drug carriers, supramolecular nanomaterials enable precise drug delivery, enhancing efficacy while minimizing toxic side effects.

Wang et al. introduced a novel therapeutic approach by developing supramolecular artificial nano‐AUTACs (GM NPs) designed to degrade the immunosuppressive enzyme IDO [155]. The core component, GN, was synthesized by conjugating the IDO ligand NLG8189 with purine‐based degradable tags. Methotrexate (MTX), a nucleoside analog, was selected for its specific affinity for GN. When mixed in a 1:1 molar ratio, MTX and GN self‐assemble through hydrogen bonding and electrostatic interactions to form nanoparticles approximately 60 nm in diameter. Intracellular acidic conditions promoted nanoparticle disassembly and drug release. MTX provided cytotoxic activity and induced autophagy, facilitating GN‐mediated IDO degradation. In tumor‐bearing mice, GM NPs reduced tumor IDO protein levels and inhibited tumor growth more effectively than free GN or the unassembled MTX–GN mixture. Pharmacokinetic analysis quantified MTX exposure and showed a half‐life of 3.832 ± 0.037 h, 1.43 times that of free MTX. The MTX AUC was also 1.55 times that of free MTX. These findings support improved in vivo efficacy and enhanced MTX exposure.

Graphene oxide (GO) is biologically flexible and an ideal material for drug delivery systems due to its 2D structure [249]. GO can be modified for TPD applications. Jin et al. constructed a nano‐APTAC platform that selectively responds to protein localization by covalently coupling protein‐specific aptamers to monolayer GO nanoparticles via amide bonding [250]. Nano‐APTAC can initiate both the autophagy–lysosomal and ubiquitin–proteasome pathways, which degrade membrane and intracellular proteins, respectively, thereby expanding the range of target proteins.

##### 4.2.4.2 Nano‐ATTEC

Because the molecular machinery linking S‐guanylation‐like tagging to K63‐linked ubiquitination remains incompletely defined, potential off‐target effects require further evaluation [251]. ATTEC, on the other hand, directly binds LC3 and targets proteins, bypassing the need for ubiquitination [90, 252]. Xu et al. developed a pH‐responsive MSPM nanoplatform that facilitates tumor‐specific protein degradation through a dual‐functionalization design [199]. The nano‐ATTEC system is constructed by self‐assembling three distinct diblock copolymers: POI (BRD4 or AR)‐ligand, LC3‐ligand (ispinesib, IS)‐modified poly(ethylene glycol)‐b‐poly(ε‐caprolactone) (PEG‐*b*‐PCL), and pH‐responsive PAE‐b‐poly(ε‐caprolactone) (PAE‐*b*‐PCL). At physiological pH, cellular internalization of nano‐ATTEC is minimal. However, in the mildly acidic environment of tumors, the PAE component becomes hydrophilic and acquires a positive charge, significantly enhancing the uptake of the nano‐ATTEC construct. The system facilitates autophagosome‐mediated degradation by bridging LC3 and the target protein through dual ligands. Additionally, it induces LC3‐II expression and promotes autophagosome formation, creating a positive feedback loop that enhances autophagy and accelerates degradation. Consequently, BRD4 degradation efficiency is notably improved.

Furthermore, Liu et al. employed bioorthogonal chemistry to achieve spatiotemporal precision in controlling ATTEC within tumors, enabling the selective depletion of mitochondria in tumor tissues [93]. Mitochondrial degradation is triggered by the tumor‐specific accumulation of Apt‐Cu30, an aptamer‐guided copper nanocatalyst. Apt‐Cu30 consists of a thymine‐rich sequence that forms copper nanoparticles (CuNPs), a MUC1/AS1411 aptamer for cancer targeting, and a linker connecting both elements. Upon localization, a bioorthogonal reaction between alkynyl‐tagged LC3‐recruiting warhead (alk‐DP) and azido‐tagged ligand of mitochondria (azi‐TPP) generates bio‐ATTECs, which induce selective mitophagy. Bio‐ATTEC induces mitochondria–LC3 interaction, tethering mitochondria to autophagosomes and triggering mitochondrial degradation in a concentration‐dependent manner. The in situ generation of bio‐ATTEC embodies the “on‐demand synthesis” strategy, circumventing systemic toxicity. It is synthesized exclusively at the tumor site, leaving normal cells largely unaffected due to the absence of aptamer‐mediated catalyst enrichment. Nano‐ATTEC resolves off‐target issues associated with ATTEC by utilizing an environmentally responsive delivery system, while bioorthogonal catalysis and aptamer‐metal nanoparticles confer spatiotemporal control over its precise activation.

##### 4.2.4.3 Nano‐CMA

CMA selectively identifies proteins with KFERQ‐like motifs via the chaperone HSC70 [110]. These proteins then interact with the lysosomal membrane protein LAMP2A, leading to their degradation in the lysosome [253]. CMA has therapeutic potential for treating tumors and neurodegenerative diseases by modulating key signaling pathways, including Wnt/β‐Catenin, p300/NF‐κB/NLRP3, and cGAS–STING [254, 255, 256]. Persistent activation of STAT3 is closely linked to the initiation and progression of various cancers, but traditional STAT3 inhibitors face challenges in clinical application due to poor selectivity and off‐target toxicity. To address this, Song et al. developed a CMA‐targeting chimera (CMATAC) comprising a STAT3‐binding peptide and an HSC70‐binding peptide connected by a linker [195]. CMATACs were subsequently encapsulated in LNPs (nCMATACs), which were further modified with an IGF2R‐targeting peptide to generate InCMATACs for tumor‐targeted delivery. When combined with a fasting‐mimicking diet, the expression of IGF2R and CMA pathway activity in tumor cells was increased, thus enhancing the anti‐tumor effect. In a similar vein, Wang et al. proposed a SM‐CMADs, offering a universal solution for lysosome‐mediated protein degradation [117]. They utilized the self‐assembly properties of the biphenylglycine (δδRR) motif to mix target protein ligands (such as ERα, AR, MEK1/2, and BCR–ABL) with KFERQ peptides in a controlled ratio. This strategy demonstrated promising degradation effects across various cancer models.

The integration of nanotechnology has significantly expanded the potential of AUTAC/ATTEC and CMA systems. Incorporating external stimulus‐response mechanisms into nano‐AUTAC/ATTEC/CMA platforms represents a critical step toward achieving precise, spatiotemporal degradation of target proteins. However, further advancements are necessary to optimize this approach for greater efficacy and clinical applicability.

## 5 Clinical Development and Translational Progress

Although the mechanistic and delivery repertoire of TPD has expanded substantially, clinical translation has progressed unevenly across modalities. Preclinical animal studies establish the feasibility and biological consequences of degradation across different pathways, while clinical studies evaluate its therapeutic relevance in patients. The therapeutic translation of TPD depends on whether target depletion can be reproduced in vivo and ultimately translated into clinical benefit. This section summarizes current progress from preclinical validation to clinical development.

### 5.1 Preclinical Animal Evidence

Animal studies provide an important intermediate step between mechanistic proof of degradation and clinical development. For UPS‐directed degraders, in vivo studies have established that target depletion can be linked to pathway suppression and therapeutic responses. Vepdegestrant produced ER degradation and tumor growth inhibition in ER positive breast cancer models, while degradation of KRAS^G12D^ and simultaneous depletion of BCL‐xL and BCL‐2 extended this principle to oncogenic drivers and antiapoptotic dependencies [10, 257, 258]. Nanotechnology can further modify in vivo exposure and tissue delivery. Sequentially responsive nano‐PROTACs, for example, enhanced tumor penetration and prolonged CDK4/6 degradation in colorectal tumor models [9].

Lysosomal degradation has expanded the accessible target space beyond intracellular proteins. ASGPR‐directed PCSK9 degradation altered lipid metabolism in hyperlipidemic mice, whereas VEGF and 5‐HT_2A_ receptor‐directed LYTACs showed therapeutic activity in retinal angiogenesis and hepatic fibrosis models, respectively [145, 259, 260]. Receptor‐mediated lysosomal degradation of SIRPα has also been applied to cardiac repair after myocardial infarction [171]. Autophagy‐directed approaches provide complementary access to aggregation prone proteins. Engineered exosome delivery promoted α‐synuclein clearance in Parkinson disease models, while TauAutac‐3 reduced pathological Tau and associated lysosomal stress in Alzheimer disease models [209, 261]. Together, these studies demonstrate that targeted degradation can produce pharmacodynamic and disease related effects in vivo across distinct cellular pathways. We further summarize representative in vivo studies across these degradation pathways, together with their associated pharmacodynamic and disease related outcomes (Table 3).

**TABLE 3:** Representative preclinical animal evidence.

Pathway and modality	Target	In vivo model	Target degradation and disease related evidence	References
UPS/PROTAC	ER	ER positive breast cancer xenograft and resistant models	Vepdegestrant induced tumor ER degradation and inhibited tumor growth. Combination studies with CDK4/6 or PI3K/mTOR pathway inhibitors further evaluated therapeutic responses.	[257]
UPS/dual target PROTAC	BCL‐xL and BCL‐2	Small cell lung cancer xenograft models	The dual degrader 753b depleted BCL‐xL and BCL‐2 and produced tumor growth delay or regression.	[10]
UPS/PROTAC	KRAS^G12D^	KRAS^G12D^ mutant xenograft models	ASP3082 produced selective KRAS^G12D^ degradation, suppressed downstream signaling, and induced antitumor responses in vivo.	[258]
UPS/sequentially responsive nano‐PROTAC	CDK4/6	CT26 colorectal tumor model	pH and cathepsin B responsive nanoparticles increased tumor penetration and intracellular delivery, resulting in enhanced and prolonged CDK4/6 degradation.	[9]
Endocytosis‐lysosome/ASGPR‐directed LYTAC	PCSK9	Hyperlipidemic mice	Hepatocyte‐targeted cyclic peptide LYTACs promoted ASGPR‐dependent lysosomal degradation of PCSK9 and improved lipid related metabolic readouts.	[259]
Endocytosis‐lysosome/aptamer LYTAC	VEGF	Pathological retinal angiogenesis models	VED LYTAC promoted M6PR‐mediated lysosomal degradation of extracellular VEGF and reduced retinal neovascularization and vascular leakage.	[145]
Endocytosis‐lysosome/bifunctional LYTAC	5‐HT_2A_ receptor	Experimental hepatic fibrosis	LY‐res‐3 induced ASGPR‐dependent 5 HT_2A_ receptor degradation in hepatocytes while retaining receptor antagonism in activated hepatic stellate cells, resulting in antifibrotic activity.	[260]
Endocytosis‐lysosome/biomimetic nano degrader	SIRPα	Acute myocardial infarction model	Receptor‐mediated internalization promoted lysosomal degradation of macrophage SIRPα, enhanced efferocytosis, and improved cardiac repair.	[171]
Autophagy/engineered exosome delivered chimeric peptide	α‐Synuclein	Parkinson disease mouse model	Engineered exosomes delivered an autophagy targeting peptide and promoted autophagy lysosome‐dependent clearance of α‐synuclein aggregates.	[209]
Autophagy/AUTAC	Tau	Alzheimer disease mouse models	TauAutac‐3 promoted lysosomal clearance of Tau and reduced Tau‐associated lysosomal stress and autophagy dysfunction.	[261]

### 5.2 Clinical Development

The progression of TPD from preclinical proof of concept to human studies has been most pronounced for UPS‐directed small molecule degraders. Among these, heterobifunctional degraders have established the most advanced clinical pipeline, while molecular glues and extracellular degradation strategies are also expanding into broader therapeutic settings. According to publicly available sources, a total of 53 registered clinical trials involving targeted protein degraders were identified, covering both oncology (*n* = 46) and nononcology indications (*n* = 7) (Table 4).

**TABLE 4:** Representative PROTAC degraders in clinical development.

Drug	Principal indication(s)	Status	Sponsor	Lead NCT
**Oncology (*n* = 46)**				
Vepdegestrant	ER+/HER2−, ESR1‐mutated advanced/metastatic breast cancer	Approved	Pfizer	NCT05654623
BMS‐986365	Metastatic castration‐resistant prostate cancer (mCRPC)	Phase III	Bristol Myers Squibb	NCT06764485
BGB‐16673	Chronic lymphocytic leukemia/small lymphocytic lymphoma (CLL/SLL) and other relapsed/refractory B‐cell malignancies	Phase III	BeOne Medicines	NCT06973187
ASP3082	KRAS^G12D^‐mutant pancreatic ductal adenocarcinoma (PDAC)	Phase III	Astellas Pharma	NCT07409272
ASP3082	KRAS^G12D^‐mutant metastatic non‐small cell lung cancer (NSCLC)	Phase III	Astellas Pharma	NCT07566052
NX‐5948	CLL/SLL and other B‐cell malignancies	Phase II	Nurix Therapeutics	NCT07221500
Luxdegalutamide	mCRPC	Phase I/II	Novartis	NCT05067140
BMS‐986458	Relapsed/refractory non‐Hodgkin lymphomas	Phase I/II	Bristol Myers Squibb	NCT06090539
DT2216	Relapsed/refractory hematologic malignancies and selected solid tumors	Phase II	Dialectic Therapeutics	NCT06620302
HP518	mCRPC	Phase I/II	Hinova Pharmaceuticals	NCT06155084
HRS‐5041	Advanced prostate cancer/mCRPC	Phase I/II	Jiangsu Hengrui Pharmaceutical	NCT06568094
HRS‐1358	ER+/HER2− advanced breast cancer	Phase II	Jiangsu Hengrui Pharmaceutical	NCT06555068
HP‐568	ER+/HER2− advanced breast cancer	Phase I/II	Hinova Pharmaceuticals	NCT06757335
MT‐4561	Advanced solid tumors	Phase I/II	Mitsubishi Tanabe Pharma	NCT06943521
AZD9750	Metastatic prostate cancer	Phase I/II	AstraZeneca	NCT07336446
GSK5471713	Advanced prostate cancer	Phase I/II	GSK	NCT07332455
LRK‐4189	MSS colorectal cancer and other solid tumors; initial human studies	Phase I/II	Larkspur Biosciences	NCT07498725
ARV‐806	KRAS^G12D^‐mutant pancreatic, colorectal, lung and other solid tumors	Phase I/II	Arvinas	NCT07023731
CFT1946	BRAF V600‐mutant solid tumors	Phase I/II	C4 Therapeutics	NCT05668585
CG001419	NTRK‐driven tumors; clinical pharmacology/pain‐related studies	Phase I/II	Cullgen	NCT07394374
ABBV‐101	B‐cell malignancies	Phase I	AbbVie	NCT05753501
AC676	B‐cell malignancies	Phase I	Accutar Biotechnology	NCT05780034
ARV‐393	Relapsed/refractory non‐Hodgkin lymphoma (NHL); combination development in diffuse large B‐cell lymphoma	Phase I	Arvinas	NCT06393738
HSK29116	B‐cell malignancies	Phase I	Haisco Pharmaceutical	NCT04861779
HSK40118	EGFR‐mutant NSCLC	Phase I	Haisco Pharmaceutical	NCT06050980
NKT‐3964	Advanced solid tumors	Phase I	NiKang Therapeutics	NCT06586957
TQB3019	Advanced hematologic malignancies	Phase I	Chia Tai Tianqing	NCT06943677
NX‐2127	CLL and other B‐cell malignancies	Phase I	Nurix Therapeutics	NCT04830137
QLH12016	mCRPC	Phase I	Qilu Pharmaceutical	NCT05973149
GDC‐2992	Advanced prostate cancer	Phase I	Genentech/Roche	NCT05800665
UBX‐303061	B‐cell malignancies	Phase I	Ubix Therapeutics	NCT06590961
AC699	ER+/HER2− advanced breast cancer	Phase I	Accutar Biotechnology	NCT05654532
ARV‐110	mCRPC	Phase I/II	Arvinas	NCT03888612
CFT8919	EGFR L858R‐mutant NSCLC	Phase I	Betta Pharmaceuticals	NCT06641609
GLR2037	Advanced prostate cancer	Phase I	Gan & Lee Pharmaceuticals	NCT07468396
HJ‐004‐02	EGFR‐mutant NSCLC	Phase I	HeJing Medicine	NCT07361237
ARV‐6723	Advanced solid tumors/immuno‐oncology	Phase I/II	Arvinas	NCT07749586
PT0511	KRAS‐mutant advanced solid tumors	Phase I	PAQ Therapeutics	NCT07300150
LT‐010391	KRAS^G12D^‐mutant advanced solid tumors	Phase I	Leadingtac Pharmaceutical	NCT07624214
HP‐002	Relapsed/refractory B‐cell NHL	Phase I/II	Shanghai Helioson Pharmaceutical	NCT07658352
DS9051b	Advanced/metastatic adrenocortical carcinoma and mCRPC	Phase I	Daiichi Sankyo	NCT07189403
AUTX‐703	Relapsed/refractory acute myeloid leukemia and myelodysplastic syndromes	Phase I	Auron Therapeutics	NCT06846606
AXT‐1003	Relapsed/refractory NHL and EZH2‐driven malignancies	Phase I	Axter Therapeutics	NCT06484985
RNK‐05047	Advanced solid tumors and diffuse large B‐cell lymphoma	Phase I/II	Ranok Therapeutics	NCT05487170
BTX‐9341	HR‐positive/HER2‐negative advanced or metastatic breast cancer after prior CDK4/6 inhibitor therapy	Phase I	Biotheryx	NCT06515470
BG‐75098	Advanced solid tumors, including CDK4/6 inhibitor‐progressed settings	Phase I	BeOne Medicines	NCT07226349
**Nononcology (*n* = 7)**				
GT20029	Androgenetic alopecia	Phase II	Kintor Pharmaceutical	NCT06692465
KT‐621	Atopic dermatitis and other type‐2 inflammatory diseases	Phase II	Kymera Therapeutics	NCT07217015
LT‐002‐158	Moderate‐to‐severe atopic dermatitis	Phase I/II	Leadingtac	NCT06931990
AH‐001	Androgenetic alopecia	Phase I	AnHorn Medicines	NCT06927960
KT‐485	Healthy volunteers and hidradenitis suppurativa; broader inflammatory diseases	Phase I	Sanofi/Kymera	NCT07629336
KT‐579	Healthy volunteers	Phase I	Kymera Therapeutics	NCT07412288
BIIB142	Healthy volunteers	Phase I	Biogen	NCT07133828

*Note*: Data were manually curated from ClinicalTrials.gov (data cutoff: September 27, 2026).

In oncology, the clinical target spectrum of heterobifunctional degraders has expanded from hormone receptors to signaling proteins, transcriptional regulators, and mutation defined oncogenic drivers. Representative targets in clinical development include ER, AR, BTK, KRAS^G12D^, BCL6, and BCL‐xL [13, 262]. ER‐directed degradation has achieved regulatory approval, whereas representative degraders targeting AR, BTK, and KRAS G12D have entered Phase III development [263, 264]. In contrast, BCL6 and BCL‐xL degraders remain primarily in Phase I or Phase I/II evaluation [265, 266]. ER‐directed degradation has achieved the clearest clinical progress to date. The oral ER targeting PROTAC vepdegestrant (ARV‐471) received United States Food and Drug Administration (US FDA) approval for ESR1 mutated, ER positive, HER2 negative advanced or metastatic breast cancer [267]. In the Phase III VERITAC‐2 trial, vepdegestrant significantly prolonged progression free survival relative to fulvestrant in the ESR1 mutated population (5.0 vs 2.1 months), although no significant benefit was observed in the overall population [262]. As the first US FDA approved heterobifunctional PROTAC, vepdegestrant provides important clinical and regulatory validation of this therapeutic modality. Beyond ER, several other oncology targets have entered late stage clinical development. The AR degrader BMS‐986365 (gridegalutamide), the BTK degrader tacabrutideg (BGB‐16673), and the KRAS^G12D^ degrader setidegrasib (ASP3082) have all advanced to Phase III studies [263, 264, 268]. Setidegrasib has also shown antitumor activity in Phase I studies of KRAS p.G12D‐mutated non‐small cell lung cancer and pancreatic ductal adenocarcinoma, providing clinical evidence that TPD can be extended to mutation defined oncogenic drivers [269]. Together, these advances indicate that oncology focused TPD is expanding from hormone‐dependent targets toward hematologic signaling proteins and genetically defined oncogenic drivers.

TPD is also moving beyond oncology. The brain‐penetrant LRRK2 degrader ARV‐102 has entered clinical development for neurodegenerative diseases and has demonstrated central nervous system exposure and pharmacodynamic target engagement in humans [270]. In immunology, the oral STAT6 degrader KT 621 has progressed into Phase IIb studies in atopic dermatitis and eosinophilic asthma after early clinical studies demonstrated rapid and sustained STAT6 depletion in blood and skin [271]. These programs broaden the translational context of TPD toward chronic diseases, where sustained tissue exposure and long‐term tolerability become increasingly important.

Molecular glue development is undergoing a parallel transition. The accelerated approval of iberdomide in 2026 further established the clinical relevance of CRBN‐mediated neosubstrate degradation, while systematic efforts are expanding this principle toward previously unexploited target proteins [272, 273, 274]. For example, the VAV1‐directed molecular glue degrader MRT 6160 has entered Phase II evaluation in Sjogren's disease, illustrating the expansion of rationally developed molecular glues into immune‐mediated disorders [275, 276]. At the same time, clinical degradation is no longer restricted to intracellular proteins. Canonical LYTACs remain preclinical, whereas related ASGPR‐engaging extracellular degrader platforms have entered clinical development. Biohaven's MoDE degrader BHV‐1300 has advanced to Phase III evaluation in Graves disease [277].

Despite the growing number of degraders entering clinical development, the current pipeline remains dominated by ubiquitin proteasome system‐directed strategies. Lysosome‐directed degradation is still less clinically advanced, even though many disease relevant proteins are extracellular or membrane associated and are therefore inaccessible to conventional UPS‐based approaches. Future development should expand lysosomal degradation beyond a limited set of receptors and tissues, with particular attention to target internalization, receptor distribution, intracellular trafficking, and efficient lysosomal delivery. Such progress could substantially broaden the clinically accessible target space of TPD.

## 6 Conclusion and Prospects

This review systematically summarizes the mechanisms, molecular determinants, delivery strategies, and therapeutic development of TPD. By integrating these interconnected aspects, we aim to establish a comprehensive framework for understanding and optimizing TPD approaches, thereby facilitating the selection of appropriate degradation modalities according to target characteristics and disease contexts. By harnessing proteasome‐dependent, endocytosis lysosome‐mediated, and autophagy‐dependent degradation pathways, TPD is transforming therapeutic intervention from conventional inhibition of protein function toward active regulation of disease‐associated protein homeostasis. These evolving strategies have substantially expanded the range of addressable targets and created new opportunities to modulate intracellular, membrane‐associated, and extracellular proteins that remain difficult to control using traditional pharmacological approaches.

However, achieving effective protein degradation requires more than simply establishing binding between a degrader and its target protein. Multiple interconnected factors, including target accessibility, productive degradation complex formation, the availability and functional status of degradation machinery, and the dynamic balance between protein degradation and resynthesis, collectively determine degradation efficiency and therapeutic outcomes. These determinants vary considerably among different TPD modalities and should therefore be incorporated as essential considerations during molecular design. Future TPD development will require a transition from solely optimizing target engagement toward systematic regulation of molecular architecture, cellular context, and degradation kinetics.

Drug delivery further increases the complexity of TPD development, as sufficient systemic exposure does not necessarily ensure that degraders reach the relevant tissues, cells, or subcellular compartments required for productive activity. Nanotechnology provides important opportunities to address these limitations. Nanocarriers can improve the formulation properties of poorly soluble molecules, enhance circulation stability, modulate biodistribution, and enable responsive release and spatiotemporal control. When combined with targeting ligands, biomimetic membrane strategies, or microenvironment‐responsive designs, nanoplatforms can further regulate where and when active degraders exert their functions. Moreover, certain nanotechnological systems serve not only as delivery vehicles but also as integrated platforms that contribute directly to target recognition and intracellular trafficking through multivalent presentation, spatial organization, and functional module integration. These capabilities create new possibilities for developing advanced TPD platforms and combination therapeutic strategies.

Nevertheless, the value of nanotechnology in TPD should ultimately be evaluated based on functional outcomes rather than delivery parameters alone. Increased cellular uptake or enhanced tissue accumulation does not necessarily translate into more efficient target degradation or improved therapeutic efficacy. Future studies should determine whether nanoplatforms can increase the bioavailability of active degraders, promote sustained target protein elimination, and ultimately improve therapeutic windows in vivo. In addition, the clinical translation of nano‐enabled TPD remains challenged by issues including nonspecific uptake, immune activation, prolonged carrier retention, altered release behavior under physiological conditions, as well as batch‐to‐batch variability, storage stability, scalable manufacturing, and quality control. Therefore, future efforts should prioritize nanodegradation systems with clearly defined therapeutic objectives, controllable structural complexity, and demonstrated clinical advantages.

Looking ahead, responsive nanoplatforms could integrate tissue targeting, sequential release, and conditional activation to improve control over degradation in diseased tissues. Protein‐based and biomimetic carriers merit further development alongside rigorous assessment of their biological fate and repeated‐dose safety. Codelivery may also enable coordinated degradation of complementary targets or removal of resistance‐associated proteins to enhance another treatment. Computational modeling and artificial intelligence could assist the joint optimization of degrader structure and carrier properties. Extending these strategies beyond cancer will require adaptation to the tissue barriers and treatment demands of neurological, inflammatory, metabolic, and cardiovascular diseases. Ultimately, combining mechanistic understanding with reliable delivery and practical manufacturing will be central to developing TPD therapies that address defined clinical needs.

## Author Contributions

Kun Zhang proposed this theme and organized the skeleton. Huaijuan Guo, Haishuang Duan, Yang Liu, Kelan Fang, Lujia Xiao, Ling Bai, and Xiao Qu reorganized figures and wrote the manuscript. Kun Zhang and Xuebing Yan revised this manuscript, supervised, and supported the project. All authors have read and approved the final manuscript.

## Funding

This work was supported by the National Natural Science Foundation of China (No. 81902422), the China Postdoctoral Science Foundation (No. 2024M762749), China Postdoctoral Science Foundation—Anhui Joint Support Program (2024T028AH), the Natural Science Foundation of Jiangsu Province (No. SBK2023022610), the program of Jiangsu Traditional Chinese Medicine Science and Technology Development (No. MS2024140), and the Program of Yangzhou Commission of Health (No. 2023‐2‐01, 2024‐2‐08).

## Ethics Statement

The authors have nothing to report.

## Conflicts of Interest

The authors declare no conflicts of interest.

## Data Availability

No primary research results, software, or code have been included and no new data were generated or analyzed as part of this review.
